# A high throughput mutagenic analysis of yeast sumo structure and function

**DOI:** 10.1371/journal.pgen.1006612

**Published:** 2017-02-06

**Authors:** Heather A. Newman, Pamela B. Meluh, Jian Lu, Jeremy Vidal, Caryn Carson, Elizabeth Lagesse, Jeffrey J. Gray, Jef D. Boeke, Michael J. Matunis

**Affiliations:** 1 Department of Biochemistry and Molecular Biology, Johns Hopkins University, Bloomberg School of Public Health, Baltimore, MD, United States of America; 2 High Throughput Biology Center and Department of Molecular Biology and Genetics, Johns Hopkins University, School of Medicine, Baltimore, MD, United States of America; 3 Department of Chemical and Biomolecular Engineering, Johns Hopkins University, Baltimore, MD, United States of America; The University of North Carolina at Chapel Hill, UNITED STATES

## Abstract

Sumoylation regulates a wide range of essential cellular functions through diverse mechanisms that remain to be fully understood. Using *S*. *cerevisiae*, a model organism with a single essential SUMO gene (*SMT3*), we developed a library of >250 mutant strains with single or multiple amino acid substitutions of surface or core residues in the Smt3 protein. By screening this library using plate-based assays, we have generated a comprehensive structure-function based map of Smt3, revealing essential amino acid residues and residues critical for function under a variety of genotoxic and proteotoxic stress conditions. Functionally important residues mapped to surfaces affecting Smt3 precursor processing and deconjugation from protein substrates, covalent conjugation to protein substrates, and non-covalent interactions with E3 ligases and downstream effector proteins containing SUMO-interacting motifs. Lysine residues potentially involved in formation of polymeric chains were also investigated, revealing critical roles for polymeric chains, but redundancy in specific chain linkages. Collectively, our findings provide important insights into the molecular basis of signaling through sumoylation. Moreover, the library of Smt3 mutants represents a valuable resource for further exploring the functions of sumoylation in cellular stress response and other SUMO-dependent pathways.

## Author summary

The Small ubiquitin-related modifier (SUMO) is a 100 amino acid protein that is attached to other proteins and thereby regulates nearly all essential cell functions. To explore how this small protein modifier functions to regulate so many different processes, we generated a library of >250 mutant alleles of the SUMO gene in the budding yeast, *S*. *cerevisiae*. Our analysis of these mutants provides the first comprehensive structure-function based map of the SUMO protein and identifies conditional mutant alleles that can be used to explore the role of sumoylation in protecting cells from proteotoxic and genotoxic stress.

## Introduction

Small ubiquitin-related modifiers (SUMOs) are ~100 amino acid proteins that are covalently attached to other proteins and thereby function as reversible, posttranslational protein modifications. Hundreds of proteins are regulated through sumoylation, accounting for effects on nearly every aspect of cell function including control of gene expression, DNA replication and repair, mRNA processing and export, mitochondrial fission, cytoskeleton assembly and signaling at the plasma membrane [1–4]. Explaining how sumoylation regulates such a wide range of proteins and processes remains an actively pursued research challenge. Some of the complexities of sumoylation in vertebrates may be explained in part by the expression of multiple SUMO paralogs, the best characterized being SUMO1, SUMO2, and SUMO3. Whereas SUMO2 and SUMO3 are ~96% identical (and therefore referred to as SUMO2/3), SUMO1 is only ~45% identical to SUMO2 and SUMO3 and may have unique signaling properties and functions [5]. In contrast to vertebrates, the budding yeast *S*. *cerevisiae* express a single SUMO gene originally identified as a high copy suppressor of a mutation in the centromere protein Mif2 and therefore named Suppressor of Mif Two (*SMT3*) [6]. Because *S*. *cerevisiae* contains a single SUMO gene, it represents an ideal model organism in which to investigate the essential functions of sumoylation and the molecular mechanisms underlying the complexities of SUMO signaling. A wealth of information concerning SUMO substrates, protein-protein interactions and genetic interactions between pathway components have also been generated through high throughput studies and contribute to the utility of *S*. *cerevisiae* as a model system [7–13].

Early genetic analysis of the SUMO pathway components in *S*. *cerevisiae* revealed essential roles for sumoylation in regulating progression through mitosis. Yeast sumoylation mutants arrest as large budded cells in metaphase and have defects in the anaphase promoting complex/cyclosome (APC/C) mediated proteolysis of securin, Pds1 and mitotic cyclins, demonstrating an essential role for sumoylation in the metaphase to anaphase transition [14–17]. In addition, mutational analysis of SUMO conjugating and deconjugating enzymes in *S*. *cerevisiae*, as well as SUMO-targeted ubiquitin E3 ligases (STUbLs), have provided critical insights into the roles for sumoylation in DNA damage repair and maintenance of genome integrity [18–21]. Recent genetic studies in yeast have also contributed important insights to an emerging view of SUMO as “molecular Velcro”, due its ability to promote protein-protein interactions [22]. This effect is mediated by the ability of SUMO to be covalently conjugated to proteins and simultaneously associate non-covalently with proteins containing SUMO-interacting motifs (SIMs) [23]. Studies in yeast have also contributed to understanding the functions of sumoylation in multiple other pathways, including transcription regulation [24–26], nuclear transport [27–29], mRNA metabolism [30, 31] and cellular stress response pathways [32].

Despite this progress, mutational analysis of the Smt3 protein itself represents an opportunity for discovery that has not been fully explored. Lysine to arginine substitution mutants of Smt3 predicted to affect chain formation have been studied, leading to the general conclusion that polymeric chains regulate normal chromatin structure but are not required for essential SUMO functions [8, 33]. In addition, two conditional mutations corresponding to amino acid substitutions in buried residues that likely disrupt Smt3 folding have also been reported [15, 34]. In an effort to develop a comprehensive structure-function based map of Smt3 and identify mutant alleles that could be used to further explore critical functions, we generated a library of 252 mutant *smt3* alleles that can be expressed and screened in *S*. *cerevisiae*. Each mutant in the library was uniquely barcoded to allow quantitative high-throughput analysis of complex phenotypes. Through an initial screening of this mutant library, we identified 12 lethal *smt3* alleles as well 45 alleles with conditional growth phenotypes that can be used to further explore the roles of sumoylation in cellular stress response pathways. Our studies provide a comprehensive analysis of the Smt3 protein and insights into the molecular basis of signaling through sumoylation.

## Results

### Design of a versatile episomal/integratable synthetic *SMT3* cassette

**Fig 1:**
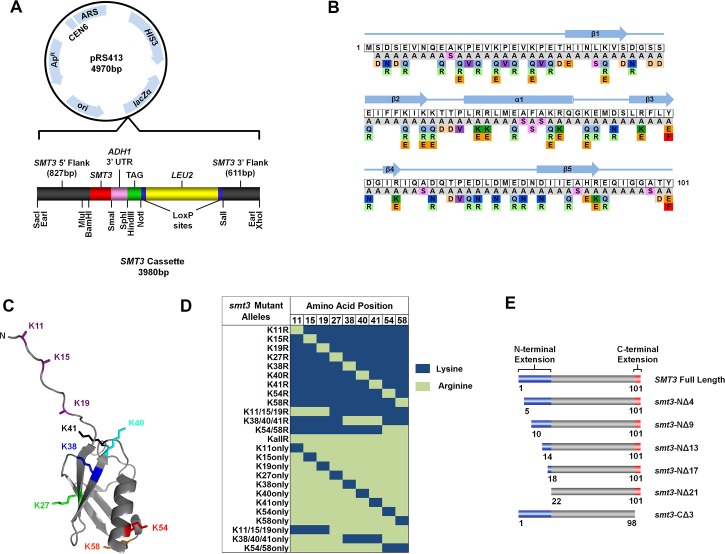
Development of a versatile library of yeast SUMO mutants. (A) Schematic illustration of the *SMT3* base construct within the pRS413 vector. Positions of the 5’ and 3’ *SMT3* flanking regions, *SMT3*, *LEU2*, *ADH1* 3’UTR, useful restriction sites and the TAG region, are shown. (B) Illustration of the amino acid substitutions present in the *SMT3* mutant collection. Individual wild type residues (in white boxes) were substituted with residues shown directly below. (C) The 9 lysine residues in Smt3 mapped onto the Smt3 crystal structure (PDB: 1EUV). (D) Table summarizing the lysine to arginine substitutions included in the *SMT3* mutant collection. (E) Summary of N- and C-terminal deletions included in the *SMT3* mutant collection.

In order to identify residues of yeast SUMO critical for its many essential functions, we developed a library consisting of >250 *smt3* mutant alleles. As a first step in the construction of the *SMT3* mutant collection, a *SMT3* cassette that would be used for the generation of each mutant was created. The *SMT3* cassette was based on a previously described synthetic cassette used for the generation of a histone mutant library and was designed to increase the versatility of the final *SMT3* mutant collection [35]. The *SMT3* cassette was synthesized by Bio Basic Incorporated (Canada) and cloned into the pRS413 vector (Fig 1A). The cassette contains ~1400 base pairs of sequence flanking the 5’ and 3’ ends of the *SMT3* open reading frame that allow the mutant collection to be expressed using the natural *SMT3* promoter as well as allowing integration of the mutant alleles into the endogenous *SMT3* gene locus. The pRS413-*SMT3* construct contains two selectable markers, *HIS3* and *LEU2*. While either marker allows for expression of the mutant collection from a CEN (episomal single copy) vector, the *LEU2* marker adjacent to *smt3* allows for selection of integrated mutant alleles and therefore expression from the endogenous *SMT3* gene locus. The *LEU2* marker is flanked by LoxP sites to facilitate its Cre-dependent removal following integration or exchange with any other marker flanked by LoxP sites. Another important feature of the *SMT3* cassette is that it contains a “TAG” region that would allow complex phenotypes of the mutant collection to be analyzed by microarray. The “TAG” region consists of a unique pair of barcodes for each mutant flanked by universal primer sequences. Finally, numerous restriction enzyme sites were engineered into the *SMT3* cassette in order to easily exchange sections of the cassette as needed.

### *SMT3* mutant library

The *SMT3* mutant library consists of 252 uniquely bar-coded mutants of the *SMT3* gene. Each mutant was synthesized by as a 600 base pair section of the original *SMT3* cassette that was unique in the *SMT3* and “TAG” regions. In order to probe the functionality of each Smt3 residue, every residue, including surface residues as well as those predicted to be buried, was mutated as illustrated in Fig 1B. Each residue was mutated to alanine while all alanine residues were mutated to serine. To neutralize the charge of acidic residues, aspartic and glutamic acid residues were mutated to asparagine and glutamine, respectively. Likewise, lysine residues were mutated to glutamine. To reverse charges, both aspartic and glutamic acid were mutated to arginine while lysine and arginine were mutated to glutamic acid. Tyrosine residues were mutated to phenylalanine to probe for dependence on the tyrosyl moiety. To probe for effects of potential post-translational modifications, all residues that can be phosphorylated, including serine, threonine, tyrosine and histidine, were mutated to aspartic (serine and threonine) or glutamic (tyrosine and histidine) acid to mimic a constitutively phosphorylated state. Lysines were mutated to glutamine and arginine to mimic the acetylated and deacetylated states, respectively. Prolines were mutated to valine to eliminate proline isomerization and arginines were mutated to lysine to prevent arginine methylation. Finally, the two previously identified conditional mutations, L26S and F52S, were also included as controls [15, 34]. Since a substitution mutant at a single residue may not be sufficient to induce a phenotype, several multi-site mutants were created in regions that are highly conserved between the yeast and human SUMOs.

Smt3 contains nine lysine residues that localize to four surface-exposed regions (Fig 1C). Smt3 lysines can serve as sites for Smt3 or ubiquitin (Ub) conjugation, as well as potential sites for acetylation or methylation. Individual and multi-site lysine to arginine mutations were therefore generated to determine the effect of losing SUMO chain formation or post-translational modification at particular lysine residues (Fig 1D). To further probe the functionality of chain linkage or modification at particular sites, either single or multiple lysines were added back to a lysine-less mutant, KallR.

In addition to substitution mutations, deletion mutants of the N- and C-termini of Smt3 were also included in the library (Fig 1E). The N-terminal extension of Smt3 is twenty-one amino acids in length and is absent in other ubiquitin-related proteins. This extension contains three SUMO consensus motifs (ΨKXE/D) that are the major sites of chain formation [33]. To test the functionality of the entire N-terminal region as well as each of the SUMO consensus motifs, six N-terminal deletion mutants were included in the mutant collection. In addition to the N-terminal extension, Smt3 also contains a three amino acid C-terminal extension that is removed by the SUMO protease, Ulp1, to reveal the terminal G98 that is essential for conjugation to substrate lysines [36]. Since regulating the processing of the Smt3 C-terminal extension could be a way to control the level of Smt3 available for conjugation, a C-terminal truncation mutant was included in the library to determine the functional significance of losing precursor processing. Lastly, the library also included human SUMO1, SUMO2 and SUMO3 to determine the functionality of each of the different human paralogs in yeast.

All of the mutants were integrated into the *SMT3* gene locus of a *smt3*Δ strain harboring wild-type *SMT3* on a *URA3* based shuffle plasmid. Previous work has shown that mutants in the SUMO pathway can have amplified levels of the 2μm circle plasmid that lead to slow, cold-sensitive growth and cell cycle delays [37]. In order to identify mutant phenotypes that are distinct from those that arise from 2μm hyper-amplification, the *smt3*Δ shuffle strain used to construct the mutant collection was cured of the 2μm circle plasmid prior to mutant integration.

### Human SUMOs

**Fig 2:**
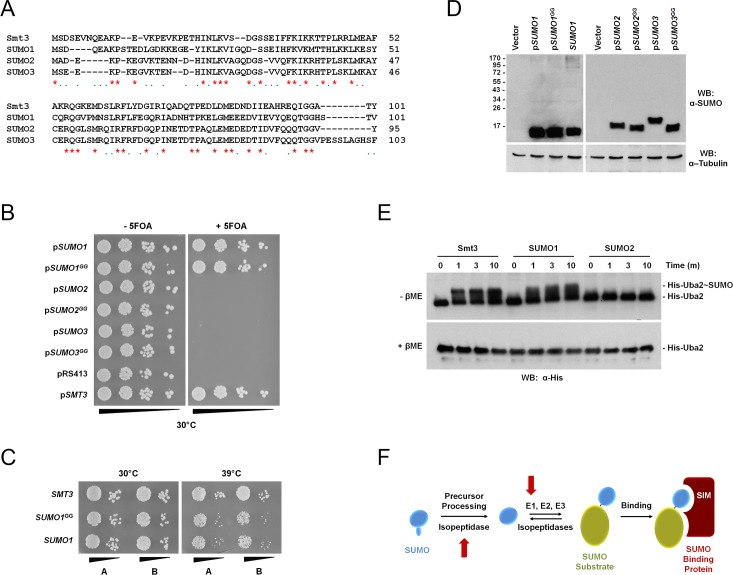
Analysis of *SMT3* deletion complementation by human SUMOs. (A) Sequence alignments between Smt3 and human SUMO paralogs. (B) SUMO1, but not SUMO2 or SUMO3, complement *SMT3* deletion. A *smt3*Δ strain harboring a copy of wild-type *SMT3* on a *URA3*-based plasmid (pRS315) was transformed with the indicated plasmids (*pSUMO*) containing a *HIS3* selectable marker. The cells were serially diluted and spotted onto histidine minus plates in the absence or presence of 5-FOA and cultured at 30°C. (C) SUMO1 expressing strains are temperature sensitive. Strains containing integrated constructs encoding *SMT3*, *SUMO1* precursor or *SUMO1* mature protein (*SUMO1*^*GG*^) were grown at 30°C and 39°C for 2 days. (D) Immunoblot analysis of SUMO expression and conjugation in wild type yeast transformed with the indicated plasmids (*pSUMO*) or in a strain containing integrated SUMO1 (*SUMO1*). (E) SUMO2 is not activated by the *S*. *cerevisiae* E1 activating enzyme. Purified, recombinant *S*. *cerevisiae* His-Uba2/Aos1 E1 heterodimer was incubated with Smt3, SUMO1 or SUMO2 in the presence of ATP. Reactions were stopped at the indicated time points by addition of SDS sample buffer with or without β-mercaptoethanol (βME). Uba2~SUMO thioester intermediates were detected by immunoblot analysis. (F) Schematic illustrating the precursor processing and E1 activation defects (red arrows) associated with SUMO2 expression in *S*. *cerevisiae*.

Human SUMO1, SUMO2 and SUMO3 each share ~50% sequence homology with yeast *SMT3* (Fig 2A). To address the functionality of human SUMO1, SUMO2 and SUMO3 in *S*. *cerevisiae*, we designed three gene constructs encoding human SUMO1, 2 and 3 based on our original *SMT3* cassette. To optimize expression of the human constructs in our *smt3Δ* strain, each human SUMO cDNA was re-coded with yeast-optimized codons using the Codon Juggling module of the GeneDesign software [38]. In addition, each human SUMO paralog was encoded as both the precursor and mature form of the protein in order to eliminate defects due to precursor processing by the yeast SUMO isopeptidase, Ulp1. Plasmids encoding the human SUMOs were transformed into the *smt3Δ* strain harboring wild-type *SMT3* on a URA3-based CEN plasmid. Transformants were then plated onto synthetic complete media lacking histidine in the absence or presence of 5-FOA at 30°C to select for cells that had lost the wild-type *SMT3* containing plasmid. Previous work had shown that SUMO1 could complement a *smt3Δ* strain [39]. Similarly, we found the human SUMO1 precursor and mature proteins were able to complement the *smt3Δ* strain (Fig 2B). However, neither the precursor nor the mature forms of SUMO2 or SUMO3 were able to complement the *smt3Δ* strain (Fig 2B). Although both the precursor and mature forms of human SUMO1 could complement the deletion strain at 30°C, both strains displayed increased sensitivity to a variety of stress conditions including heat, DNA damage, cadmium chloride and oxidative stress (Figs 2C and 4D).

In order to understand why human SUMO2 and SUMO3 could not compensate for the loss of Smt3, the protein expression of all of the human SUMOs was analyzed by western blotting using antibodies specific for the human SUMO paralogs. The human SUMOs were expressed in a wild-type strain also expressing Smt3. SUMO1 was expressed and also appeared to be conjugated to substrates as it was visible in high molecular mass smears in the *SMT3* shuffle strain (Fig 2D). Since both SUMO1 and Smt3 were expressed in the strain, competition between the two proteins could prevent robust substrate conjugation by SUMO1. To determine if SUMO1 and Smt3 were in competition for substrate conjugation, we also profiled the expression of the SUMO1 integrated strain that lacked expression of Smt3. Consistent with competition, the high molecular mass SUMO1 smears increased in the integrated strain that lacked Smt3 expression. Notably, the level of human SUMO1 conjugates in the integrated strain appeared modest compared to the level of free SUMO1 and may contribute to the stress sensitivity of the human SUMO1 strains (Fig 2D). Like SUMO1, SUMO2 and SUMO3 precursor and mature proteins were expressed in the *SMT3* shuffle strain (Fig 2D). However, both the SUMO2 and SUMO3 precursors failed to be processed to the mature forms of the proteins based on their electrophoretic mobilities (Fig 2D). Furthermore, neither SUMO2 nor SUMO3 appeared to be competent for conjugation since the mature forms of both proteins were not detected in high molecular mass conjugates.

Since the defects associated with the mature forms of SUMO2 and SUMO3 were not due to expression defects, we examined the first step of the SUMO conjugation pathway, activation and E1 thioester formation. For these experiments, purified recombinant Smt3, SUMO1 or SUMO2 were incubated with the His-tagged *S*. *cerevisiae* Uba2p/Aos1p E1 heterodimer. The samples were incubated in the presence of ATP and E1 thioester formation was monitored by non-reducing and reducing SDS-PAGE and anti-His western blots. Both Smt3 and SUMO1 formed a thioester with His-Uba2p; however, SUMO2 did not (Fig 2E). Our findings indicate that, whereas SUMO1 complements *smt3Δ* under normal growth conditions, SUMO2 and SUMO3 do not due to defects in both precursor processing and E1 activation (Fig 2F).

### Identification and analysis of lethal *smt3* mutant alleles

**Fig 3:**
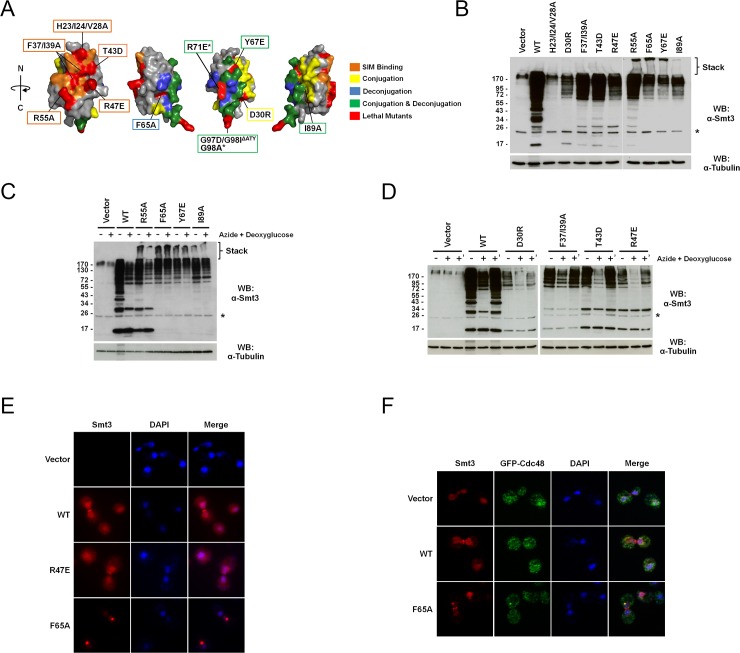
Analysis of lethal *smt3* mutant alleles. (A) The identified lethal mutations mapped onto the Smt3 crystal structure (PDB: 1EUV). Mutated residues giving rise to lethal phenotypes are highlighted red. Also highlighted are residues predicted or known to be important for conjugation (yellow), deconjugation (blue), conjugation and deconjugation (green) or SIM binding (orange). Lethal mutations in residues important for conjugation, deconjugation or SIM binding are boxed with appropriate corresponding colors. *R71E and G98A mutations were episome remedial. (B) Analysis of the expression and conjugation profiles of lethal *smt3* mutant alleles. Mutant alleles were expressed in a SUMO1 integrated strain and then analyzed by immunoblot analysis. The asterisk and high molecular mass species seen in the vector only control (also in C and D) represent non-specific, cross-reacting proteins. (C) Analysis of deconjugation in response to ATP depletion. Lethal mutant alleles that form ultra-high molecular mass conjugates were expressed in a SUMO1 integrated strain. Cultures at mid-log phase were grown in normal medium (-) or ATP depletion medium (+) containing sodium azide and 2-deoxyglucose for 10 minutes. Cell lysates were analyzed by immunoblot analysis. (D) Analysis of deconjugation and conjugation following ATP depletion and restoration. The lethal mutants not forming ultra-high molecular mass conjugates were expressed in a SUMO1 integrated strain. Cultures at mid-log phase were grown in normal (-) or ATP depletion media (+) for 10 minutes. Cells were then allowed to recover for 10 minutes in normal medium (+’). Cell lysates were analyzed by immunoblot analysis. (E) Analysis of Smt3 protein localization. The indicated Smt3 proteins were expressed by transforming a SUMO1 expressing strain with the indicated constructs. Transformants were grown to mid-log phase, fixed, spheroplasted and permeabilized. Smt3 localization was determined by immunofluorescence microscopy. DNA was labeled with DAPI. (F) Co-localization of Smt3 foci and Cdc48. A GFP-Cdc48 expressing strain was transformed with empty vector or vectors coding for wild type or F65A mutant Smt3. Transformants were grown to mid-log phase and fixed, spheroplasted and permeabilized. Smt3 and GFP-Cdc48 localization were determined by immunofluorescence microscopy. DNA was labeled with DAPI.

The ability of human SUMO1 to complement loss of Smt3 indicates significant plasticity in primary amino acid sequences required for functionality. To identify residues essential for viability, the ability of individual mutants in the library to survive in the absence of Smt3 was determined by a plasmid shuffle technique [40]. Mutants that failed to produce plasmid-free segregants when integrated into the endogenous *SMT3* gene locus were initially considered lethal. Using this approach, 15 mutant alleles failed to support growth in the absence of wild-type *SMT3* (Fig 3; S1A Fig). Three of the identified alleles, F37/I39/T42A, R55/Q56A and L81/E84/D87/I89A, were multi-site mutants that overlapped with already represented lethal alleles and were excluded from the final lethal mutant list.

Previously described work with a synthetic histone collection showed that 25% of the lethal mutants identified by integration were episome remedial presumably due to multiple plasmid copies [35]. To determine if the lethal *smt3* alleles could also be remediated by episomal expression, each mutant was also expressed from a centromeric plasmid. Only two alleles, R71E and G98A, were episome remedial. Thus, 10 alleles failed to complement growth of the *smt3Δ* strain whether expressed from the *SMT3* genomic locus or a plasmid and were characterized further (S1A Fig).

A defect in precursor processing could provide a simple explanation for the lethality of the identified Smt3 mutants. Each lethal mutant was therefore expressed as the mature form of the protein and 5-FOA resistance of each mutant in the *smt3Δ* shuffle strain was assessed. Like their full-length counterparts, none of the mature, lethal mutants were viable in the absence of wild-type *SMT3* (S1B Fig). However, two of the mutants, F37/I39A and T43D, did give rise to weak 5-FOA resistant growth after extended incubation. Since lethal mutations may also cause protein instability and reduced expression, each lethal mutant was also expressed on a 2μm plasmid in the *smt3Δ* shuffle strain. Overexpression of the mutants also failed to suppress lethality and in some cases, overexpression led to a dominant negative phenotype (S1B Fig).

**Fig 4:**
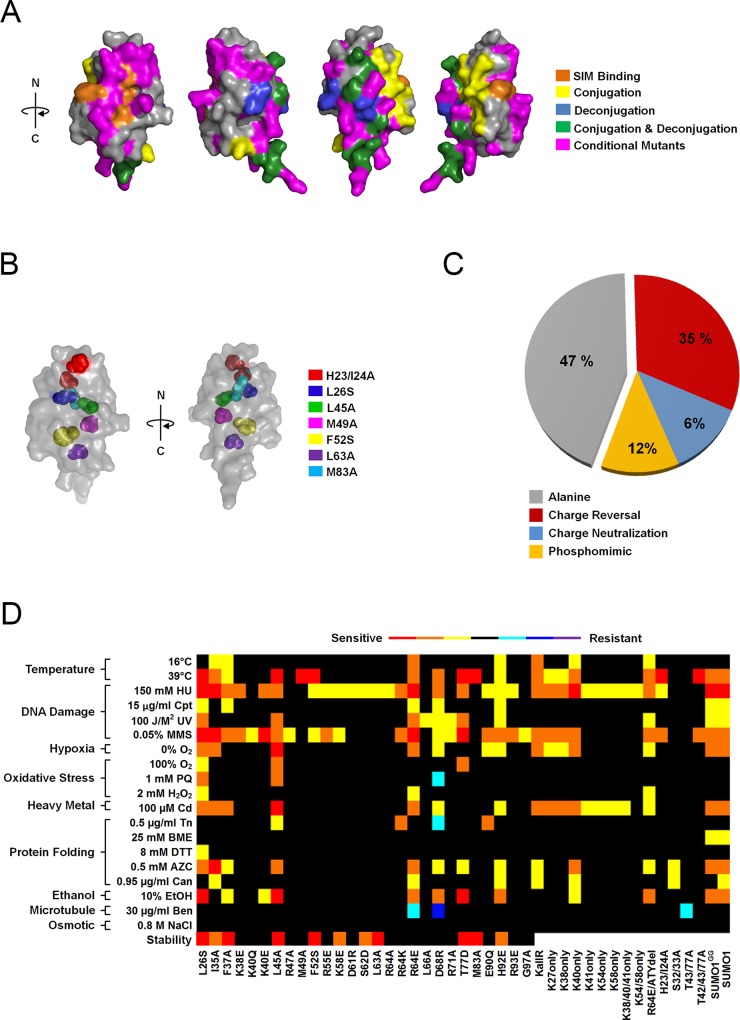
Analysis of *smt3* conditional mutant alleles. (A) The identified conditional mutations mapped onto the crystal structure of Smt3 (PDB: 1EUV). Residues that gave rise to conditional phenotypes are marked with magenta. Also highlighted are residues predicted or shown by other studies to be important for conjugation (yellow), deconjugation (blue), conjugation and deconjugation (green) and SIM binding (orange). (B) Conditional mutations in residues that are either completely or partially buried were mapped onto the Smt3 crystal structure. (C) Analysis of the percentage of conditional mutants identified according to the type of substitution made at a given residue. (D) A heat map representing the growth phenotypes of the *smt3* conditional alleles (indicated at the bottom) under the indicated growth conditions (indicated on the left). The absence of a phenotype is marked with black while increasing sensitivity is marked with yellow, orange and red, respectively. Similarly, increasing resistance is marked with cyan, blue and purple, respectively. Predicted effects of mutations on protein stability based on Rosetta analysis are also indicated. Black indicates mutations predicted to be neutral or stabilizing (REU (Rosetta Energy Unit) < 1.0), while increasing instability is marked with orange (REU between 1.0–2.0) and red (REU > 2.0).

To better understand the defects associated with the lethal alleles at the molecular level, each mutation was mapped on the structure of Smt3 together with residues previously identified through biochemical and crystallographic studies as important for SIM and SUMO pathway component interactions (Fig 3A and Table 1) [23, 41–45]. Since mutating a buried residue might reduce protein stability and lead to lethality, we first assessed whether any of the identified lethal mutations map to buried residues in the wild type Smt3 structure. Of the lethal mutations, the side chain of one, I89A, is predicted to be buried, while several mutants including H23/I24/V28A, F37/I39A, F65A and Y67E, correspond to partially buried residues (Fig 3A; S2A Fig). To further explore possible effects of these mutations on Smt3 stability, we used the macromolecular modeling software Rosetta to estimate changes in the protein stability for single point mutants in our library [46, 47]. Based on this analysis, each mutant was classified as destabilizing or neutral/stabilizing (Fig 4D, S2 Table). Notably, the lethal mutations at each of the buried or partially buried residues were predicted to have destabilizing effects, suggesting that defects in Smt3 folding or stability may contribute to their lethal phenotypes.

**Table 1:** Summary of predicted interactions between lethal and conditional Smt3 amino acid substitutions and the E1 activating enzyme, E2 conjugating enzyme, Ulp1/2 isopeptidases (only structures of Ulp1 have been formally determined) and SIMs of downstream interacting proteins. Substitutions predicted to be destabilizing based on Rosetta analysis are indicated in bold (REU (Rosetta energy unit) > 2.0) or underlined (REU between 1.0–2.0) text. **Predicted interactions are based on published, structure-based analysis of human or yeast protein complexes, as summarized in the final row.

	E1 Interaction	E2 Interaction	Ulp1/2 Interaction	SIM/E3 Interaction	Core Residues
**Lethal Mutations**	R71E, G98A, G97D/G98I^ATYdel^	**D30R**, **Y67E**, **I89A**	**F65A**, **Y67E**, R71E, **I89A**, G98A	**F37/I39A**, T43D, R47E, R55A	**H23/I24/V28A**, **F37/I39A**, **F65A**, **I89A**
**Conditional Mutations**	D61R, R71A, E90Q, H92E	D68R, E90Q, H92E	R64A, R64K, R64E, R64E^ATYdel^, L66A, D68R, R71A, R93E, G97A	I35A, **F37A**, K38E, K40Q, K40E, R47A, R55E, T42/T43/T77A	**L26S**, I35A, **F37A**, **L45A**, **F52S**, **L63A**, **M83A**
**Structure-Based Smt3 Interactions**	S32, D61, R71, E90, H92, E94, Q95, G97, G98	K27, S29, D30, G31, Y67, D68, I70, D82, E84, N85, D87, I88, I89, E90, H92	R64, L66, Y67, D68, G69, I70, R71, Q73, Q76, D82, E90, H92, R93, E94, Q95, I96, G97, G98	I35, F36, F37, K38, I39, K40, T43, R47, L48, A51, R55	L26, V28, S29, I35, F37, I39, L45, L48, F52, L63, F65, I72, P78, M83, I89, A91
	**Predicted from human complex: [41]	**Based on yeast complexes: [45,52]	**Based on yeast and human complexes: [42, 61]	**Based on yeast complex, Smt3 modeling and predictions from human complexes: [44,52,55]	**Based on Smt3 structure: [42]

Although F65 and Y67 are partially buried, these residues are also part of an important interaction surface for Ulp1 binding, suggesting a possible defect in isopeptidase recognition [42]. Other lethal mutations, including F37/I39A, T43D, R47E and R55A, map to regions at or near the SIM binding surface on Smt3 (Fig 3A). SIMs are critical for interactions between SUMO and E3 ligases, thus effecting substrate conjugation, as well as for interactions with downstream effector proteins [48–51].

The SIM binding surface on Smt3 consists of a hydrophobic groove capped by basic residues that bind hydrophobic and negatively charged residues, respectively, within a SIM (S1C Fig). T43D and R47E introduce a negative charge predicted to affect interactions with acidic or hydrophobic SIM residues, respectively [44]. Because T43D is a phosphorylation mimic, phosphorylation at this site might be a way to regulate SIM binding. Although R55A is also on the SIM binding surface, disruption of the positive charge does not seem to be the defect associated with this mutation since the charge reversal mutant, R55E, did not lead to lethality. Additional mutations, not mapping to the SIM binding surface, were also identified. The D30R mutation mapped to a surface residue of Smt3 implicated in E2 interactions, indicating a potential defect in conjugation [45, 52]. The G97D/G98I/ΔATY mutant was included as a non-conjugatable control and expected to be lethal; it maps to the extreme C-terminus of Smt3. Surprisingly, the G98A lethal mutation was episome remedial, suggesting only partial defects in processing and conjugation.

To further characterize the lethal mutants, the Smt3 protein expression and conjugation profiles of individual strains were analyzed by western blotting. Since the lethal mutants do not support viability, each mutant was expressed in a human SUMO1 expressing strain and Smt3 specific antibodies were used to detect the yeast protein (it should be noted that the effect of individual mutations on antibody recognition is uncertain). Although SUMO1 and Smt3 could potentially form mixed chains in these strains, individual mutants nonetheless displayed unique properties. The H23/I24/V28A protein, mutant in two buried residues, I24 and V28, appeared to be unstable since very little protein expression was detected (Fig 3B). The D30R, F37/I39A, T43D and R47E mutants each had expression patterns that were similar to wild-type Smt3 in that both conjugates and a pool of free Smt3 were detected (Fig 3B). However, D30R and R47E appeared to be expressed at lower levels compared to wild-type Smt3 and all lethal mutants showed some reduction in molecular mass conjugates between 34–72 kDa and in free Smt3 (at ~17 kDa). The R55A, F65A, Y67E and I89A mutants were expressed at varying levels relative to wild-type Smt3, but each of these mutants shared a unique phenotype, namely the presence of ultra-high molecular mass Smt3 smears that could be detected extending into the stacking gel (Fig 3B and 3C).

To understand the nature of the ultra-high molecular mass smears observed with R55A, F65A, Y67E and I89A mutants, *in vivo* ATP depletion experiments were performed to determine if the mutant proteins could be recognized by isopeptidases. Since Smt3 conjugation is an ATP-dependent reaction, ATP depletion prevents conjugation and allows isopeptidase mediated deconjugation of substrates to be observed; time-dependent deconjugation and recovery of conjugation can be observed *in vivo* by inhibiting and restoring ATP production (S1D Fig). Ultra-high molecular mass smears present in the R55A mutant were rapidly reduced during ATP depletion, demonstrating efficient isopeptidase recognition, while the ultra-high molecular mass smears present in F65A, Y67E and I89A mutants persisted following ATP depletion, indicating inefficient recognition by the isopeptidases (Fig 3C). In order to determine if the ultra-high molecular mass smears represented polymeric Smt3 chains, the F65A mutation was combined with another *smt3* allele, KallR, in which all of the lysines had been mutated to arginine to prevent chain formation. The F65A/KallR combination mutant failed to produce ultra-high molecular mass smears, indicating that the smears represent polymeric chains resulting from a defect in isopeptidase recognition (S1E Fig). Previous studies have shown that ultra-high molecular mass conjugates formed in Ulp2Δ strains contribute to toxicity [33]. To determine if introducing the KallR mutations suppressed the lethality of the F65A allele, the combination mutant was introduced into the *smt3Δ* shuffle strain harboring *SMT3* on a *URA3-*based plasmid. The lethality of F65A was weakly suppressed by the introduction of the KallR mutations at 25°C but not at higher temperatures (S1F Fig). ATP depletion and recovery analysis of the mutants that did not show ultra-high molecular mass smears (D30R, F37/I39A, T43D and R47E) revealed that all of these proteins were recognized by the conjugating and deconjugating enzymes (Fig 3D).

Finally, each of the lethal mutants was characterized by immunofluorescence microscopy to determine cellular localization. Wild-type Smt3 is typically enriched in the nucleus and also co-localizes with the septin ring during mitosis (Fig 3E). Lethal mutants that had Smt3 protein expression profiles similar to the wild-type *SMT3* strain, such as R47E, had a localization pattern indistinguishable from wild-type protein (Fig 3E). Lethal mutants that formed ultra-high molecular mass smears, such as F65A, also showed Smt3 protein enriched in the nucleus and faint septin ring staining. Notably, however, these mutants also frequently contained a single prominent nuclear focus (Fig 3E). Previous studies performed in *Schizosaccharomyces pombe* showed that the SUMO targeted ubiquitin ligase mutant, *slx8-29*, formed nuclear foci containing SUMO and the Cdc48 segregase [53]. To determine if Cdc48 co-localized with the foci we observed, F65A was expressed in a GFP-tagged *CDC48* strain. In cells expressing either empty vector or wild-type *SMT3*, the GFP-Cdc48 signal was diffuse throughout the cell and nuclear foci were not seen; however, when F65A was expressed, GFP-Cdc48 foci formed in the nucleus and co-localized with the Smt3 foci (Fig 3F). Our findings indicate that SIM binding and efficient isopeptidase recognition of Smt3 polymeric chains are two properties essential for Smt3 function.

### Identification of conditional *SMT3* alleles

To identify *SMT3* alleles with conditional defects, the integrated collection of 240 viable mutants was tested for growth in 19 different plate assays to probe for sensitivity or resistance to low and high temperature, DNA damage, hypoxia, oxidative stress, protein folding stress, osmotic stress, heavy metal, EtOH and microtubule perturbation. The growth assays were performed under conditions predetermined to slightly inhibit the wild-type *SMT3* strain in order to identify sensitive and resistant mutations. From this analysis, 45 conditional *smt3* mutations (~18% of the collection) were identified, of which nine are altered at one or more lysine residues and 2 correspond to either the full-length or mature version of human SUMO1. The remaining 34 alleles represented 32 different residues. Similar to the lethal mutants, the conditional mutants mapped to multiple Smt3 surfaces including those important for SIM binding, conjugation and deconjugation (Fig 4A and Table 1). In addition, 7 residues identified were predicted to be partially or completely buried (Fig 4B; S2 Fig). Mutations in 6 of these 7 residues were predicted to have potential destabilizing effects based on modelling using Rosetta analysis (Fig 4D, S2 Table). Notably, only 47% of the identified alleles were alanine substitutions and 41% of the residues represented by the conditional alleles would not have been identified in a simple alanine scan (Fig 4C). Analysis of protein expression and conjugation patterns under normal growth conditions suggested significantly reduced expression for a number of conditional mutants (L26S, D30A and H92E) compared to wild-type Smt3. However, subtler qualitative differences in the global SUMO modification patterns were detected for a majority of strains (S3 Fig; S1 Table).

To summarize the phenotypes observed, each mutant was given an assay score reflective of its sensitivity or resistance to a certain growth condition relative to wild-type *SMT3*. A heat map was generated using these scores to visualize the observed phenotypes of each mutant (Fig 4D). This heat map revealed that the majority of conditional growth mutants showed sensitivity, rather than resistance, in the growth conditions tested. In addition, ~60% of the mutants were pleiotropic in that they showed sensitivity or resistance in more than one growth condition. Mutants such as R64E and D68R showed sensitivity in numerous assays, but also displayed resistance in other assays such as microtubule inhibition. Finally, growth conditions affecting the largest number of mutant alleles were those causing DNA damage, temperature stress and heavy metal stress, while no mutant alleles were sensitive to osmotic stress caused by high salt.

### SIM binding is essential for viability and the stress response

**Fig 5:**
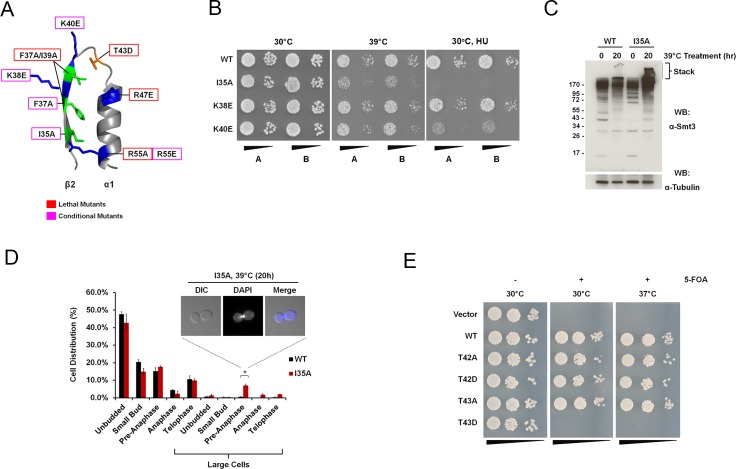
Analysis of *smt3* SIM binding mutant alleles. (A) The identified lethal and conditional *smt3* mutants found within the SIM binding surface mapped onto the β2-α1 region of Smt3 (PDB: 1EUV). Residues that gave rise to lethal and conditional phenotypes are boxed in red and magenta, respectively. (B) SIM binding mutants are stress sensitive. The indicated strains were cultured overnight and serially diluted and spotted onto plates with or without hydroxyurea (HU). The plates were incubated at 30°C or 39°C, as indicated. (C) The *smt3* I35A mutant allele accumulates unusual conjugates. Wild type and *smt3* I35A strains were grown to mid-log phase, diluted 4 fold then shifted to 39°C for 20 hrs. Samples were collected at 0 and 20 hr time points and analyzed by immunoblot analysis. The stacking portion of the gel was left intact so that ultra-high molecular mass conjugates could be visualized. (D) The *smt3* I35A mutant allele exhibits cell cycle defects. Wild type and *smt3* I35A strains were grown to mid-log phase at 30°C and then shifted to 39°C for 20 hrs. Cells were collected at 20 hrs, permeabilized and stained with DAPI. Each bar represents the average of 3 independent experiments in which at least 150 cells were counted per experiment. Vertical bars indicate the standard error. Astericks denote a p-value < 0.05. (E) Phosphorylation of T42 and T43 is not critical for *smt3* function. The *smt3Δ* shuffle strain was transformed with plasmids coding for the indicated *Smt3* alleles. The transformants were grown overnight, serially diluted and spotted onto selective media in the presence or absence of 5-FOA at 30°C and 37°C.

Many of the mutant alleles identified as either lethal or conditional mapped to the SIM binding surface on Smt3 (Fig 5A). Importantly, these mutants had varying phenotypes that could be useful in future genetic studies, including high copy suppressor analysis. Lysines 38 and 40 are of particular interest, as they are within the SIM binding surface but are located outside of the hydrophobic groove where they could interact with negatively charged regions of the SIM or with phosphorylated SIMs [54–58]. Charge reversal of K38 or K40 to glutamic acid gave rise to specific defects limited to HU and MMS sensitivity (Fig 4C and Fig 5B). In contrast, mutation of isoleucine 35, which is located within the hydrophobic groove important for interacting with hydrophobic residues within a SIM, led to numerous phenotypes including HU and MMS sensitivity, as well as weak temperature sensitivity (Fig 4C and Fig 5B). The heat sensitivity seen in I35A did not appear to be due to protein instability since western blot analysis revealed protein expression levels comparable to wild-type Smt3 at 30°C and 39°C (Fig 5C). However, the pattern of conjugated proteins seen in I35A was different at both temperatures in comparison to the pattern seen in the wild-type strain (Fig 5C). In addition, while wild-type *SMT3* and I35A both displayed increased levels of conjugates after 20 hours at 39°C, I35A had an increased level of ultra-high molecular mass conjugates observed in the stacking gel (Fig 5C). Since previous studies have shown that *smt3* mutants such as *smt3-331* and *smt3-KallR* display chromosome segregation defects [8, 15], the cell cycle profiles of wild-type *SMT3* and the I35A mutant grown at high temperature were compared. When grown at 39°C for 20 hrs, the I35A mutant gave rise to large budded pre-anaphase cells that accounted for a small subset (10.7%) of the cell population consistent with the phenotypes seen with *smt3-331* and *smt3-KallR* (Fig 5D).

Another interesting mutant identified in the SIM binding site was the lethal T43D phosphomimic (Fig 5E). Threonine 43 lies within the loop between the first alpha helix and second beta sheet in Smt3 adjacent to lysine residues important for binding negatively charged residues within a SIM [44]; therefore, it is possible that phosphorylation of this residue, or mutation to an acidic residue such as aspartic acid, could inhibit SIM binding (Fig 5A). If phosphorylation at threonine 43 serves as a means to regulate SIM binding, then it is possible that loss of this regulation would result in reduced viability. In order to test this hypothesis, T43 was also mutated to alanine to mimic a protein that could not be phosphorylated at that site. In all of the assays tested, T43A exhibited no growth defects indicating that regulation of SIM binding through phosphorylation of this site is not critical for viability under these conditions (Figs 4D and 5E). Similarly, mutation of the neighboring threonine, T42, to either aspartic acid or alanine resulted in no notable defects (Fig 4D).

### Characterizing the formation and function of Smt3 polymeric chains

**Fig 6:**
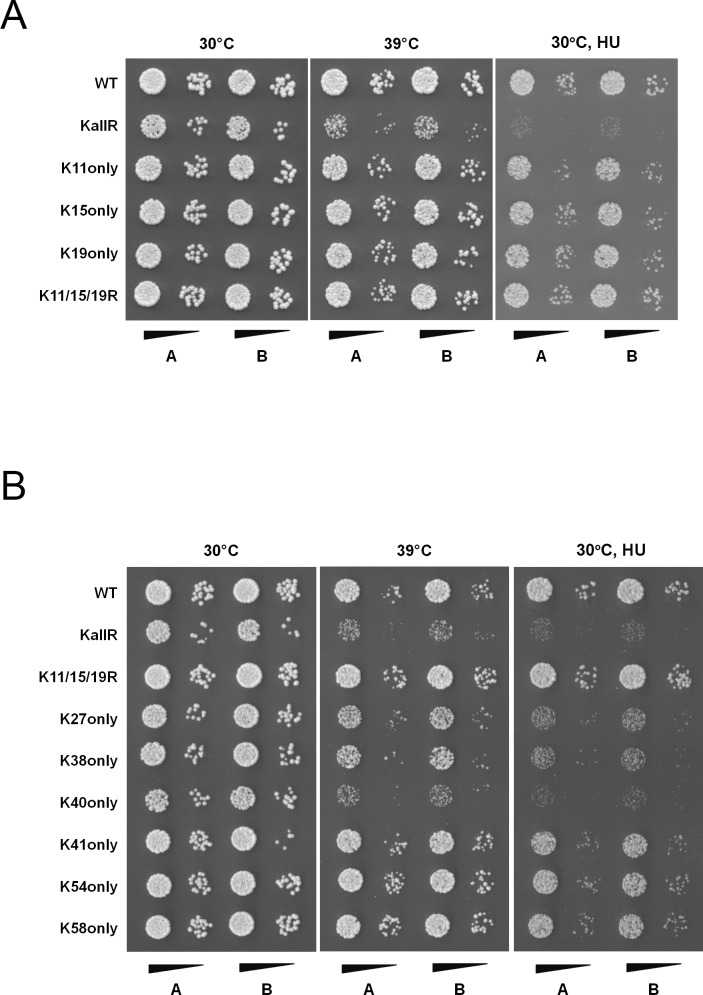
Analysis of *smt3* K/R mutant alleles. (A) and (B) Strains expressing the indicated *smt3* alleles were grown overnight, serially diluted then spotted onto plates in the presence or absence of HU. The plates were incubated at 30°C or 39°C, as indicated.

Like ubiquitin, Smt3 contains multiple lysine residues that could serve as sites for synthesis of topologically and functionally distinct chains (Fig 1C). Three of the lysines at amino acid positions 11, 15 and 19 are within modification consensus motifs; moreover, lysine 15 appears to be the major site of chain formation *in vivo* [33]. Previous work has shown that although chain formation through internal lysines is not essential for viability in *S*. *cerevisiae*, cells expressing a version of Smt3 that contains no lysines (KallR), have an increased doubling time, chromosome segregation defects and sensitivity to agents that cause DNA damage and replication stress [8, 33]. We also found that the KallR chain forming mutant displayed sensitivity to agents that cause DNA replication stress such as HU and MMS, as well as a weak sensitivity to growth at high temperature (39°C), hypoxia, CdCl_2_ and the protein damaging agents AZC and canavanine (Figs 4D and 6A).

To investigate more specifically the functional significance of linkage-specific chains, lysine residues were restored in the KallR mutant to allow chain formation at individual positions. Consistent with previous data demonstrating that lysine 15 is the major site for chain formation, restoration of lysine at this position rescued the associated phenotypes of the KallR mutant (Fig 6A) [33]. Furthermore, restoring lysines within either of the other two modification consensus sites at amino acid positions 11 or 19, also fully rescued the phenotypes of the KallR mutant (Fig 6A). Although lysines 11, 15 and 19 restore the functionality of the KallR mutant, loss of chain formation at these sites in the K11/15/19R mutant or N-terminal deletion mutant, resulted in no detectable growth defects, demonstrating that one or more of the other six lysine residues may serve as sites for functional chain formation (Fig 6A). Restoration of individual lysines at amino acid positions 41, 54 or 58 suppressed the temperature sensitivity of the KallR mutant. However, restoration of lysines at amino acid positions 27, 38, 41, 54 or 58 only partially suppressed HU-induced DNA damage sensitivity (Fig 6B). Notably, restoration of lysine 40, a lysine located within the SIM binding site, did not suppress the temperature or HU sensitivity of the mutant, indicating that chain synthesis through this lysine is either inefficient or that chains formed at this site are unable to function in these stress responses (Fig 6B). These results indicate that while chain formation through internal lysine residues is not essential for viability, it is important for optimal response to certain stress conditions, including replication stress, high temperature, heavy metal stress, hypoxia and protein folding stress. Furthermore, chain formation at lysines 11, 15 and 19 are the preferred sites for modification under the conditions tested.

## Discussion

We have developed a versatile library of >250 mutant alleles of *S*. *cerevisiae* SUMO (Smt3) using biochemical gene synthesis technologies. Owing to rapidly declining synthesis costs over the past decade [59], this approach was efficient and economical, and it allows for additional synthetic mutants to be easily generated and added to the collection. As demonstrated, the library can be screened using traditional colony-forming assays and also has the potential to be screened using high-throughput approaches that take advantage of individual barcodes associated with each mutant allele. Through our screens and analysis, we have generated a comprehensive structure-function based map of Smt3 and residues that are critical for viability and responses to a variety of cellular stresses. Importantly, our analysis identified 45 conditional *smt3* alleles that can be used to further investigate the molecular mechanisms underlying effects of sumoylation on stress survival.

### Human SUMO complementation

Although only ~50% identical to Smt3, human SUMO1 was previously shown to complement a *smt3*Δ strain [39]. Our findings with both the precursor and mature forms of SUMO1 validated these results. Because homology is distributed evenly across the full length of Smt3 and SUMO1, complementation suggests a considerable degree of plasticity built into the SUMO structure and interactions with factors required for conjugation, deconjugation and downstream signaling. Nonetheless, plate growth assays used to test the response to different stress conditions showed that strains expressing either the precursor or mature form of SUMO1 were hypersensitive to various stresses. These sensitivities correlated with relatively the low levels of high molecular mass conjugates detected in these strains (despite robust SUMO1 expression), indicative of an imbalance between rates of conjugation and deconjugation. Consistent with low conjugation levels affecting fitness, strains lacking the major Smt3 E3 ligase, Siz1, exhibit a similar imbalance in conjugation and deconjugation rates and similar stress sensitivities [60]. Notably, the lack of growth defects in both SUMO1 and Siz1Δ strains under normal growth conditions also suggests that viability can be maintained with diminished levels of SUMO conjugates [60].

Unlike SUMO1, all of the SUMO2 and SUMO3 constructs that we tested failed to complement the *smt3*Δ strain. SUMO2 and SUMO3 were expressed, but were not conjugated to proteins due to defects in precursor processing and E1 activation. The observed defects in precursor processing are consistent with in vitro studies demonstrating that the C-terminal tails of SUMO2 and SUMO3 precursors are incompatible with Ulp1 recognition and cleavage [61]. Although differences in E1 recognition are not as readily explained, it is noteworthy that residues of SUMO1 important for human E1 interaction are more highly conserved with Smt3 (7 of 11 residues) than with SUMO2 and SUMO3 (only 3 of 11 residues) [41]. Although phylogenetic studies indicate that SUMO1 and SUMO2/3 gene families arose early in metazoan evolution, the question of whether SUMO1 or SUMO2/3 more closely resemble the single ancestral SUMO of lower eukaryotes remains uncertain [62]. Taken together, our observed functional differences suggest that SUMO1 may be more closely related evolutionarily to Smt3 than SUMO2 and SUMO3.

### Functional surfaces on Smt3

The screens that were performed on the *smt3* mutant collection identified 12 lethal and 45 conditional alleles. Notably, the entire 21 amino acid N-terminal extension of Smt3 could be deleted without detectable effects on growth under the conditions assayed. The function of the N-terminal extension, present in SUMO proteins from yeast to human, therefore remains unclear. Similarly, we observed no consequences of deleting the C-terminal extension of the Smt3 precursor protein. Why SUMO, ubiquitin and Nedd8 proteins are all synthesized as precursors in organisms from yeast to human, again remains unclear. Mutant alleles that did exhibit growth defects represented mutations in residues that mapped to two distinct surfaces on the Smt3 structure. One surface comprised the “backside” and C-terminal tail that make important contacts with conjugating and deconjugating enzymes. Lethal mutations mapping to this surface included F65A, Y67E and I89A, which may affect interactions with the Ulp1 and/or Ulp2 isopeptidases either directly or through indirect effects on structural stability. Analysis of these mutants in SUMO1 expressing strains revealed that they are expressed and processed by Ulp1, the dominant processing isopeptidase [36], and accumulate as high molecular mass conjugates resistant to deconjugation following ATP depletion. These mutations may therefore more severely affect recognition by Ulp2, whose loss also results in formation of toxic, high molecular mass conjugates [33, 63]. Lethal mutations mapping to this surface, including D30R, Y67E, and I89A may also affect non-covalent interactions with the backside of Ubc9 thought to be important for E3 ligase function, polymeric chain synthesis, or recruitment of Ubc9 to sumoylated substrates and protein complexes [45, 50, 52]. Mutations in Ubc9 that affect this non-covalent interaction with Smt3 are inviable [45], consistent with a role in promoting essential functions.

The second critical surface on Smt3 identified in our screens mediates interactions with the major class of SIMs present in E3 ligases and interacting proteins functioning downstream of conjugation. Structural studies have shown that these SIMs, consisting of a hydrophobic core flanked on either side by negatively charged residues, interact with a common surface formed by the alpha helix and second beta strand on SUMO [43, 44, 48, 55, 64–66]. We identified multiple lethal mutations within the SIM binding surface, consistent with an expected essential role for non-covalent SUMO-SIM interactions. Of the lethal mutations, two are predicted to affect interactions with the SIM hydrophobic core (F37/I39A and R47E), and two are likely to disrupt formation of stabilizing interactions with acidic or phosphorylated residues (R55A and T43D) [44, 55]. Thus, these mutations reinforce the importance of both hydrophobic and electrostatic contacts in forming functionally stable SUMO-SIM interactions. It should be emphasized that defects in SIM binding may affect both substrate modification, through defects in E3 activity [50], as well as binding to downstream effector proteins. Defects in protein stability, as suggested by Rosetta modelling analysis, and reduced expression levels of some of these mutants may also contribute to their lethal phenotypes.

The conditional alleles with mutations mapping to these two surfaces of Smt3 are of particular interest due to their potential utility in further exploring the roles of sumoylation in genotoxic and proteotoxic stress response pathways. A number of these mutants, including R64E, D68R, R71A and G97A are predicted to affect Ulp1 recognition [61], and consistent with this prediction these mutants accumulate unprocessed precursor protein (S3 Fig). However, these mutants also accumulate near normal levels of free processed Smt3 and protein conjugates, suggesting that conditional growth defects may be more directly related to Ulp1- or Ulp2-dependent deconjugation of specific substrates. Notably, Ulp2-deficient strains are also hypersensitive to DNA damaging agents similar to the R64E, D68R, R71A and G97A mutants [33, 67]. Further proteomic and genetic studies with these specific mutants could prove valuable in identifying SUMO conjugates and affected pathways critical for DNA damage and other stress responses.

Multiple conditional mutant alleles predicted to affect SIM binding were also identified. Among these mutants, I35A and F37A are predicted to affect binding to the conserved hydrophobic core common to all SIMs [44], and these mutants exhibited broad and overlapping sensitivities to multiple stress conditions. In contrast, mutant alleles predicted to affect interactions with more variable acidic or phosphorylated residues surrounding the SIM hydrophobic core (K38E, K40Q and K40E, R55E) exhibited growth defects more tightly restricted to DNA damaging agents. This suggests the possibility that SIMs present in vital DNA repair factors contain unique acidic features or are potentially phosphorylated in response to DNA damage. Alternatively, these mutations may inhibit the modification of specific DNA repair factors by affecting interactions with Siz1 or other SIM-containing E3 ligases. High copy suppressor screens and other genetic studies are currently being performed with a number of these alleles to identify functionally important interacting proteins.

Notably, many of the identified conditional mutant alleles exhibited overlapping sensitivities to different stresses, and no individual surface on Smt3 emerged as being uniquely important for a specific stress response. However, it is important to note that many of the assay conditions tested could activate overlapping stress response pathways, thus limiting the ability to identify function-specific surfaces.

### Requirements for Smt3 polymeric chains

Ubiquitin can form chains through conjugation to its N-terminus and 7 internal lysines residues, and multiple studies indicate that chains of varying linkages have unique functions [68]. For example, chain formation through lysine 48 is essential for protein degradation while formation through lysine 63 gives rise to conditional, DNA damage sensitivities [69, 70]. Previous studies exploring Smt3 chain function found that a KallR mutant, in which all lysines are mutated to arginine, was slow growing and exhibited a variety of defects caused by aberrant higher order chromatin organization, transcription activation and DNA repair [8, 33]. Similarly, chain-forming mutants of *S*. *pombe* SUMO are viable but exhibit abnormal cell morphology and sensitivity to DNA replication stress [71]. Consistent with these findings, we found that the Smt3 KallR mutant strain is viable, but hypersensitive to numerous stress conditions.

Importantly, we also found that mutating any individual lysine in Smt3 resulted in no detectable phenotypes under the assay conditions tested. Thus, we obtained no evidence for linkage-specific polymeric chain functions as observed in the ubiquitin system. Consistent with this, reintroducing individual lysines in the KallR mutant at positions K11, K15 and K19 fully restored Smt3 function, while reintroducing lysines at K41, K54 and K58 restored all functions except minor sensitivities to cadmium and HU. Single lysines at positions K38 and K40 were less effective at suppressing KallR phenotypes, possibly due to their important roles in SIM recognition that may be disrupted through chain linkages at these positions. K27 was also largely ineffective at suppressing most KallR phenotypes. This lysine is uniquely positioned in the central core of Smt3 and may form chains inefficiently or with a geometry not recognized by downstream effector proteins such as the Slx5/Slx8 STUbL [20]. Taken together, our results indicate that while the potential to form polymeric Smt3 chains is important for optimal growth under a variety of stress conditions, chains formed through multiple different lysines appear to be functionally equivalent. It should be noted that our analysis does not formally discriminate between a requirement for Smt3-Smt3 polymeric chains in stress responses and Smt3-Ub hybrid chains (see below).

### Posttranslational modifications of Smt3

In addition to functioning as a posttranslational modification, SUMO itself can be modified by phosphorylation, acetylation and ubiquitylation. Phosphorylation has been detected at four sites in Smt3 (S2, S4, S32 and S33), and also at N-terminal serines in human SUMO1 and *S*. *pombe* SUMO [71–73]. The functional significance of these modifications, however, remains uncertain. Although our collection contained alanine and aspartic acid substitutions at all serine and threonine residues, to inhibit or mimic phosphorylation respectively, we obtained no clear evidence that phosphorylation of Smt3 affects cell growth under our specific assay conditions. Similarly, we observed no significant effects of inhibiting or mimicking lysine acetylation at any of the nine lysines in Smt3 (by arginine or glutamine substitutions, respectively), despite evidence that acetylation of lysine residues located in the SIM binding surface of human SUMOs inhibits binding to specific SIM-containing proteins [74]. Finally, regarding ubiquitylation, STUbLs have the ability to conjugate ubiquitin onto SUMO to produce hybrid SUMO-ubiquitin chains that affect protein turnover and localization [20, 75, 76]. Although the functions of hybrid chains in *S*. *cerevisiae* have not been directly analyzed, the intimate link between Smt3 function and the Slx5/Slx8 STUbL implies important roles [77–81]. Our analysis of Smt3 lysine mutations argues against a requirement for linkage-specific SUMO-Ub hybrid chains, at least for the conditions that we assayed. However, as noted above, our analysis of lysine mutations does not formally discriminate between requirements for Smt3-Smt3 polymeric chains and Smt3-Ub hybrid chains. Determining whether ubiquitin modification of Smt3 may be a critical component of cellular stress responses is an important question that will therefore require more detailed analysis.

### Comparisons between Smt3 and ubiquitin

Multiple mutagenic studies have been performed in *S*. *cerevisiae* to identify and characterize essential residues and properties of ubiquitin. A review of these studies and comparisons with our Smt3 findings reveals a number of common themes and important distinctions. An early alanine scan of Ub surface residues [69] and more recent high throughput mutagenesis studies [82, 83] identified an essential “hydrophobic patch” on the surface of ubiquitin that mediates non-covalent interactions with the majority of ubiquitin-binding proteins functioning downstream of ubiquitylation [84]. Thus, mutagenesis of both ubiquitin and Smt3 reveals that binding to downstream effector proteins is a critical determinant of function and that this is largely mediated by a single surface on each protein. Notably, however, the hydrophobic patch of Ub is not conserved in Smt3 and the α-helix and β-strand that mediates SIM binding maps to the opposite face of the Smt3 protein. Thus, ubiquitin and Smt3 have evolved to interact with distinct effector proteins using unique surfaces. A second essential surface of Ub identified in mutagenesis studies includes residues of the tail domain which are critical for conjugation and deconjugation [69, 82, 83]. While conjugation and deconjugation are also essential for Smt3 function, our findings revealed that tail residues other than the di-glycine motif are tolerant of substitutions, suggesting greater flexibility in recognition of Smt3 by conjugating and deconjugating enzymes. Alternatively, the greater sensitivity of tail domain substitutions in ubiquitin may be reflective of constraints imposed by the need to interact with a much broader range of E2 and E3 conjugating enzymes and isopeptidases relative to Smt3.

In addition to surface residues, mutagenesis studies have also revealed important roles for core residues in determining ubiquitin structure and function [82, 83, 85, 86]. Alanine substitutions in ubiquitin at I30 and L43 are lethal, and it has been proposed that L43A mutant proteins are degraded by the proteasome together with modified substrates due to reduced structural stability [86]. We also identified lethal core residue substitutions, including F65A (equivalent to ubiquitin L43), F37/I39A, H23/I24/V28A and I89A that were predicted to be destabilizing by Rosetta modeling analysis. Although the Smt3 F65A mutant accumulated in high molecular mass conjugates resistant to deconjugation, making it distinct from ubiquitin L43A, the H23/I24/V28A mutant displayed some similar properties, including reduce levels of conjugates and free protein. It is currently not known whether SUMO is degraded by the proteasome together with substrates or recycled similar to ubiquitin. Further analysis of the H23/I24/V28 mutant could provide valuable insights. Core ubiquitin residues at or near important binding surfaces were also found to be more sensitive to substitutions, suggesting that subtle structural defects may affect binding [83]. Several potentially destabilizing Smt3 core residue mutations also corresponded to residues near binding surfaces, including F37/I39A near the SIM binding surface, and F65A and I89A near Ulp1 and Ubc9 binding surfaces, suggesting a common theme. More detailed analysis of Smt3 core residue mutations and their effects on protein structure and dynamic stability will be required to more fully understand their effects on function.

In summary, we have developed a versatile library of *smt3* mutants that can be used to interrogate the functions of sumoylation in the budding yeast, *S*. *cerevisiae*. Based on structural analysis, we have provided plausible interpretations of defects associated with individual mutants that are intended to serve as starting points for more detailed molecular and cellular studies. This preliminary analysis revealed essential functions for non-covalent interactions with SIM-containing proteins and the importance and redundancy of polymeric chains linked through internal lysines. We have demonstrated the utility of this library by identifying >40 conditional mutant alleles sensitive to a variety of cellular stress conditions. It can be anticipated that future characterization of these mutants, and alternative screens of the library, will reveal new and fundamentally important insights into SUMO functions.

## Materials and methods

### Construction of the bacterial and yeast SMT3 library

Prior to library construction, the 2μm plasmid was evicted from the SMT3 shuffle strain (a derivative of S288C), *Mat****a*** *his3Δ1 leu2Δ0 met15Δ0 lys2Δ0 ura3Δ0 smt3*::*kanMX* [*pRS316*, *SMT3 CEN-URA3]*, using a dominant negative allele of Flp recombinase. In short, the SMT3 shuffle strain was transformed with pRS413-GAL10-*flp*-H305L (CEN *HIS*) (gift from Dr. Erica Johnson, Thomas Jefferson University). Transformants were passaged on synthetic media containing 2% galactose four times then were grown under non-selective conditions to isolate cells that had segregated the pRS413-GAL10-*flp*-H305L plasmid. In order to confirm that the 2μm plasmid had been evicted, PCR assays targeting two genes found on the 2μm plasmid, *FLP1* (Flp1 F: 5’ GGTGCTTGTTCGTCAGTTTGTG and Flp1 R: 5’ GACAATATCGAAACTCAGCGAATTGC) and *REP1* (Rep1 F: 5’ GCCAGAGGATGGCGAAC and Rep1 R: 5’ GCTCGCGTTGCATTTTCG), were performed.

Mutant constructs were synthesized and cloned into pRS413 by BioBasic Incorporated (Canada) and supplied as bacterial stocks. To generate the integrated mutant library, plasmids were isolated and digested with *EarI* to release the mutant construct from the pRS413 backbone. Linearized constructs were integrated into the 2 μm-less *SMT3* shuffle strain with leucine selection. Colonies were replica printed and transformants that were *LEU+*, *ura-*, G418 sensitive and 5-FOA resistant were isolated. Two independent colonies were selected for each construct and frozen in 96-well plate formats identical to the original collection. Lethal mutants that could not segregate pRS316 [*SMT3 URA*] were frozen separately in 96-well plates. For plasmid remediation studies, the 2 μm-less *SMT3* shuffle strain was transformed with mutant constructs within the intact pRS413 backbone with leucine selection.

### Library validation

As a first step in library validation, each mutant construct that was synthesized by BioBasic Inc. was sequenced verified and analyzed by restriction digests (BamHI/NotI/XhoI and BamHI/HindIII/XhoI) to ensure the mutant fragment had been cloned properly into pRS413. 100% sequence identity and proper cloning were required to pass quality control. Upon receipt of the mutant library, ~20% of the mutant plasmids were isolated and analyzed by restriction digests to ensure the identity of each mutant within the well. Finally, the integrated constructs within every strain in the Smt3 library were PCR amplified (*SMT3*–874 F: 5’ GCACCTATAACTCTCAACTTTGAAG 3’ and *LEU2* R: 5’ CGAATTTGATTCTGTGCGATAGC 3’) to ensure proper targeting of the construct and all PCR products were sequence verified.

### Growth media

Synthetic complete (SC) growth media for culturing yeast was prepared with 0.67% (w/v) yeast nitrogen base without amino acids (US Biological), 2% (w/v) glucose, 2% (w/v) bacto-agar supplemented with 2% (w/v) amino acids. Strains were cured of *URA3*-based plasmids by culturing on synthetic media containing 1 mg/ml 5-fluoroorotic acid (5-FOA) [87]. YPD was prepared with 2% (w/v) bacto-peptone, 1% (w/v) yeast extract, 2% (w/v) glucose and 2% (w/v) bacto-agar. Luria broth for culturing bacteria was prepared with 1% (w/v) bactopeptone, 0.5% (w/v) yeast extract and 0.5% (w/v) NaCl supplemented with either 50 μg/ml carbenicillin (CARB), 34 μg/ml chloramphenicol (CAM) and/or 50 μg/ml kanamycin (KAN).

### E1 thioester assay

The *S*. *cerevisiae* E1 activating enzyme expression constructs were provided by Dr. Chris Lima (Memorial Sloan Kettering Cancer Center) as pET15b-*AOS1* (CARB) and pET28b-HIS-*UBA2* (KAN). The E1 constructs were co-expressed in *E*. *coli* BL21 Rosetta cells. SUMO1, SUMO2 and Smt3 were all expressed as N-terminally tagged GST proteins in BL21 Codon Plus cells. The N-terminal GST tag was removed by digestion with Precission protease after purification. E1 thioester formation was assayed in reactions containing 0.0225 μg/μl E1, 0.0375 μg/μl SUMO or Smt3, 5 mM MgCl_2_, 20 mM Hepes pH 7.5 and 50 mM NaCl. Thioester formation was initiated with 5 mM ATP. Samples were collected at various time points then were combined with an equal volume of 2X sample buffer (0.125 M Tris-HCl pH 6.8, 4% SDS, 20% glycerol, bromophenol blue) with or without 1.43 M beta-mercaptoethanol (β-ME). The samples were separated by SDS-PAGE and proteins were transferred to 0.2 μm PVDF membrane (Immobilion, BioRad) overnight at 4°C (100 volts, 350 mAmps). Membranes were analyzed by incubation with a monoclonal antibody to detect the His epitope (GE Healthcare Life Sciences) followed by chemiluminescent detection (SuperSignal West Pico substrate—Thermo Scientific).

### Phenotypic growth assays

Cells were grown overnight in 100 μl of synthetic complete media in 96-well plates at 25°C. Overnight cultures were serial diluted 25-fold. For SC plates, 8 μl of two dilutions (1:625, 1:15,625) was spotted onto the test plates. For YPD plates, 2.5 μl of two dilutions (1:625, 1:15625) was spotted onto the test plates. Spots were allowed to dry on the bench at 25°C for 30 minutes before the plates were incubated.

For heat sensitivity experiments, plates were incubated at 25°C, 30°C, 34°C, 37°C and 39°C for 3 days. For cold sensitivity experiments, plates were incubated at 16°C for 2 weeks. For drug studies, SC plates were supplemented with 150 mM hydroxyurea (HU), 0.05% methylmethanesulfonate (MMS), 1.5 mM hydrogen peroxide (H_2_O_2)_, 1 mM paraquat (PQ), 100 μM CdCl_2_, 10% EtOH, 0.8 M NaCl, 8 mM dithiothreitol (DTT) and 0.5 mM Azetidine-2-carboxylic acid (AZC). To test camptothecin (Cpt) sensitivity, SC plates were buffered with 25 mM HEPES (pH 7.2) and supplemented with 15 μg/ml Cpt. To test canavanine (CAN) sensitivity, SC–Arg plates were supplemented with 0.95 μg/ml canavanine. For β-ME experiments, YPD media was supplemented with 25 mM β-ME. For tunicamycin (Tn) experiments, YPD media was supplemented with 0.5 μg/ml Tn. Benomyl (Ben) sensitivity was tested on YPD media containing 30 μg/ml benomyl. For UV experiments, cells were spotted onto YPD plates, irradiated using a UV Stratalinker (Invitrogen) and incubated in the dark. For all of the aforementioned drug and UV studies, plates were incubated at 30°C for up to 7 days. Plates were photographed at 2 and 3 days as well as at later time points between 5 and 7 days, as appropriate. For hypoxia and hyperoxia experiments, SC plates were supplemented with 15 mg/L ergosterol and 0.5% Tween-80. For hypoxia experiments, the plates were incubated in an anaerobic workstation (Coy Laboratory) equilibrated to 30°C for 3 days. For hyperoxia experiments, plates were placed in a chamber that was flushed with 100% oxygen for 30 minutes. After the chamber was sealed, the chamber containing the plates was placed in an incubator equilibrated to 30°C for 3 days. Plates for the hypoxia and hyperoxia experiments were photographed at 3 days. All assays were scored at the 3 day time point using a 5-point scale ranging from -3 to +1 with -3 being the most sensitive, 0 representing growth equivalent to wild-type *SMT3* and +1 representing resistance. Heat maps were generated by comparing the scores of each strain under the control and test conditions.

### Structure mapping

Mutant residues identified in this study as well as other mutagenic studies with ubiquitin and Nedd8 were mapped onto the surface of their corresponding protein, Smt3 (1EUV: ChainB), Ubiquitin (1UBQ) or Nedd8 (1NDD: Chain B), using Pymol [42, 69, 88–90].

### Immunoblot analysis

Cells were grown overnight to saturation in synthetic media at 30°C. The cells were diluted to an OD_600_ of 0.25 in 10 mls of synthetic media the following day and were allowed to grow to an OD_600_ of 0.8. Given that the SUMO profile does change during different growth phases, all OD_600_ measurements were monitored closely. The cells were harvested at 1,500 x g for 3 minutes then washed with 1 ml of fresh medium. The wash step was performed with fresh medium since SUMO conjugation is rapidly lost in PBS. The supernatant was removed and cell pellets were immediately frozen on dry ice and stored at -80°C. For temperature shift experiments, cells were grown to an OD_600_ of 0.8 then shifted to 37°C or 39°C for varying times between 3 and 20 hours. For the 20 hour shift experiments, the cultures were diluted 1:5 in pre-warmed media before the temperature shift to prevent saturation of the culture.

Whole cells lysates were prepared by resuspending the cell pellets in 200 μl of 20% trichloroacetic acid (TCA). The pellets were lysed by bead beating. After the bead beating, 1 ml of 5% TCA was added to the pellets and the samples were left on ice for 10 minutes. The TCA precipitated samples were centrifuged at 15,000 x g for 5 minutes and the supernatant was removed. The TCA pellets were resuspended in 2X sample buffer (0.125 M Tris-HCl pH 6.8, 4% SDS, 20% glycerol, 1.43 M β-ME, 0.04 M Tris base, 0.02% bromophenol blue). The samples were boiled for 5 minutes, centrifuged at 15,000 x g for 5 minutes to pellet insoluble debris and the supernatants were used as the whole cell lysate.

The cell lysates were then separated by SDS-PAGE on either a 12.5% or 15% acrylamide gel. Proteins were transferred from the gel to 0.2 μm PVDF membrane (Immobilion, BioRad) overnight at 4°C. Membranes were blocked in 5% milk and washed with 1X TS (0.05 M Tris base, 0.135 M NaCl, 0.32% v/v HCl) supplemented with 0.05% Tween20. Primary antibodies used included mouse anti-SUMO1 (21C7, 1:1000), mouse anti-SUMO2/3 (8A2, 1:800), rabbit anti-Smt3 (affinity purified against recombinant Smt3, 1:200) and rabbit anti-tubulin 2 (1:20,000). All antibodies were diluted in 1X PBS supplemented with 2% bovine serum albumin (BSA) and were incubated with PVDF membranes for 1 hour at 25°C. Anti-mouse and Anti-rabbit HRP-linked secondary antibodies (Amersham, 1:3,000) were diluted in 5% milk and incubated with membranes for 1 hour at 25°C. Membranes were analyzed by chemiluminescent detection (ECL plus substrate, Amersham).

### ATP depletion assays

Cells were grown overnight to saturation in synthetic media at 30°C. The cells were diluted to an OD_600_ of 0.25 in synthetic media the following day and were allowed to grow to an OD_600_ of 0.8 in a final volume of 10 mls. The cells were harvested at 1,500 x g for 3 minutes, washed with 1 ml of fresh medium and then resuspended in 1 ml of fresh medium. At this point 333 μl of the sample was removed, the cells were harvested and the pellet was frozen on dry ice. This was considered the “Start” sample. The remaining 667 μl of cells were washed with 1X PBS, harvested, then resuspended in 1 ml of ATP depletion solution (10 mM sodium azide, 10 mM 2-deoxyglucose in 1X PBS). Cells were incubated in the ATP depletion solution at 30°C for anywhere between 30 seconds and 30 minutes. After the depletion, 500 μl of the sample was removed, cells were harvested and the pellet was frozen on dry ice. This was considered the “Deplete” sample. The remaining 500 μl of cells were washed with 1ml of 1X PBS, harvested and resuspended in 1 ml of pre-warmed synthetic medium. Cells were allowed to recover at 30°C for anywhere between 30 seconds and 10 minutes. Following recovery, the cells were harvested and the pellet was frozen on dry ice. This was considered the “Recover” sample.

### Immunofluorescence

Cells were grown overnight to saturation in synthetic media at 30°C. The cells were diluted to an OD_600_ of 0.25 in synthetic media the following day and were allowed to grow to an OD_600_ of 0.8 in a final volume of 10 mls. The cells were fixed by incubation with 0.1 volumes of 37% formaldehyde for 1 hour at 25°C with gentle rocking. After fixation the cells were washed with 1 ml KSorb (0.1 M KPO_4_, 1.2 M Sorbitol) three times. All centrifugation steps were performed at 500 x g for 3 minutes. The cells were spheroplasted by resuspending the washed cell pellet in 1 mL of KSorb supplemented with 0.14 M β-ME and 7.5 mg/ml zymolyase 20T (US Biological). The cells were rocked gently on a nutator at 25°C for 20 minutes (~50% phase dark). The spheroplasts were washed gently two times in 1 ml KSorb then stored on ice. The spheroplasts were placed on a poly-lysine coated slide and allowed to settle for 1 hour in a humidity chamber at 25°C. The supernatant was aspirated from the slide then the slide was transferred to -20°C methanol for 6 minute and -20°C acetone for 30 seconds. The slide was allowed to air dry for at least 2 minutes then the slide was blocked with 2% BSA in 1X PBS for 1 hour at 25°C in a humidity chamber. The block was aspirated off of the slide and primary antibody was added to the slide and incubated overnight at 4°C in a humidity chamber. Primary antibodies used included rabbit anti-Smt3 (affinity purified against recombinant Smt3, 1:200) and mouse anti-GFP (1:200, N86/6 Neuro MAb UC Davis). All antibodies were diluted in 1X PBS supplemented with 2% BSA. The slide was washed four times in 2% BSA in 1X PBS. Secondary antibody conjugated to Alexa Fluor 488 or Alexa Fluor 594 (Life Technologies, Grand Island, NY) was diluted (1:1,000 in 2% BSA in 1X PBS) and added to the slide for 2 hours at 25°C in a humidity chamber. The slide was kept in the dark for all future steps. The slide was washed four times in 2% BSA in 1X PBS and two times with 1X PBS. The slide was allowed to dry, mounting solution (100 mM Tris-HCl pH 8.8, 50% glycerol, 2.5% DABCO and 0.2 μg/ml DAPI) was added and then the slide was sealed with a coverslip. The *CDC48* strain tagged C-terminally with GFP was obtained from the Yeast GFP Clone Collection (Invitrogen).

### Microscopy for cell cycle analysis

Cells were grown overnight to saturation in synthetic media at 30°C. The cells were diluted to an OD_600_ of 0.25 in synthetic media the following day and were allowed to grow to an OD_600_ of 0.8 in a final volume of 10 mls. The cells were fixed by incubation with 0.1 volume of 37% formaldehyde for 1 hour at 25°C with gentle rocking. The cells were harvested by centrifugation at 1,500 x g for 3 minutes then were washed twice with 1 ml of 1X PBS. The cells were permeabilized with 70% EtOH on ice for 40 minutes then harvested and washed twice with 1X PBS. The cells were sonicated (7 pulses at 1.5 output / 20% duty cycle), placed on a microscope slide, mounting solution (100 mM Tris-HCl pH 8.8, 50% glycerol, 2.5% DABCO and 0.2 μg/ml DAPI) was added and the slide was sealed with a coverslip.

### Thermodynamic stability predictions

To predict the effect of each mutation on thermodynamic stability (ΔΔG), differences in energy score (Rosetta Energy Units) were calculated for each single mutant in residues 20–98 of the Smt3 crystal structure (PDB: 1EUV) using PyRosetta v3.4.0 [46, 47]. To create a baseline, all side chains in the wild type structure were repacked, minimized, and scored using the 2010 Dunbrack rotamer library and the talaris2013 score function [91]. For each mutation, 50 structures were produced and scored using the following procedure: (1) the mutation was made and any residue within 10 Å of the affected residue was repacked, (2) the backbone was then minimized, along with all side chains, and (3) the reported ΔΔG was derived from the average of these structures.

## Supporting information

S1 Fig Analysis of lethal mutants. (A) Summary of lethal alleles that failed to complement growth of the *smt3*Δ strain. Alleles in red failed to suppress both when expressed as episomal and integrated constructs. Multi-site mutations redundant with already represented alleles were not considered for further analysis. Growth after 3 days at 30°C is indicated on a four point scale ranging from no growth (-) to growth similar to wild-type *SMT3* (+++). (B) Summary of lethal alleles that were tested for complementation in the *smt3Δ* shuffle strain to determine if maturation or overexpression of the alleles from a 2 μm plasmid suppressed lethality. Growth after 3 days at 30°C is indicated on a four point scale as in panel A. Each mutant was also expressed in a wild-type *SMT3* strain to determine if any produced a dominant negative phenotype. The Smt3 profiles of each mutant expressed in a SUMO1 strain were also analyzed by immunoblotting to detect the presence of ultra-high molecular mass Smt3 conjugates running in the stacking gel. (C) Schematic representing the Smt3 SIM binding surface bound to a canonical SIM. (D) Wild-type *SMT3* cells were grown to mid-log phase at 30°C and treated with ATP depletion media for various time points, as indicated. Cells were also allowed to recover for various times after ATP depletion, as indicated. (E) A SUMO1 expressing strain was transformed with the indicated plasmid constructs. Transformants were grown to mid-log phase at 30°C in SC-His and whole cell lysates were analyzed by immunoblotting with a Smt3 antibody. The stacking portion of the gel was left intact so that ultra-high molecular mass conjugates could be visualized. (F) The *smt3Δ* shuffle strain harboring wild-type *SMT3* on a *URA3*-based plasmid was transformed with the indicated plasmid constructs. The transformants were grown at 25°C on SC–Ura–His overnight and serial dilutions were spotted onto SC–Ura–His and SC–His + 100 μg/ml 5-FOA at 25°C and 30°C to monitor growth in the absence of wild-type *SMT3*.
(TIF)

S2 Fig Analysis of core residue mutations. (A) Illustrations of the Smt3 structure highlighting the positions of core residues shielded from solvent. (B) Table summarizing the individual core mutants analyzed, their sensitivity to growth at 39°C (Ts) and the number of additional conditional growth defects detected for each mutant (see Fig 4D for a summary of specific conditional growth defects). n/a = not applicable, n.d. = none detected.
(TIF)

S3 Fig Analysis of steady-state Smt3 conjugation in conditional mutants. Two independent isolates of the indicated conditional mutant strains were cultured at 30°C. Cells were isolated at mid-log phase and analyzed by immunoblot analysis with an antibody specific for Smt3. Tubulin was also detected as a loading control. Asterisks indicate detection of unprocessed precursor protein in R64E, D68R, R71A and G97A strains.
(TIF)

S1 Table Summary of conditional Smt3 mutant interaction defects and SDS-PAGE/western blot analysis. Levels of detected Smt3 or Smt3 conjugates are indicated on a scale from undetectable (-) to ultra-high (++++).
(PDF)

S2 Table Thermodynamic stability predictions for Smt3 single point mutations. The estimated effect of single point mutations on Smt3 thermodynamic stability (ΔΔG) was determined using the Rosetta macromolecular modeling software. Average ΔΔG values, determined as described in the Materials and Methods, are shown for each mutant.
(PDF)
