# Supplementary figures and images for: A high throughput mutagenic analysis of yeast sumo structure and function
Source: PLoS Genet. 2017 Feb 6;13(2):e1006612. doi: 10.1371/journal.pgen.1006612 (PMC5319795; doi:10.1371/journal.pgen.1006612)

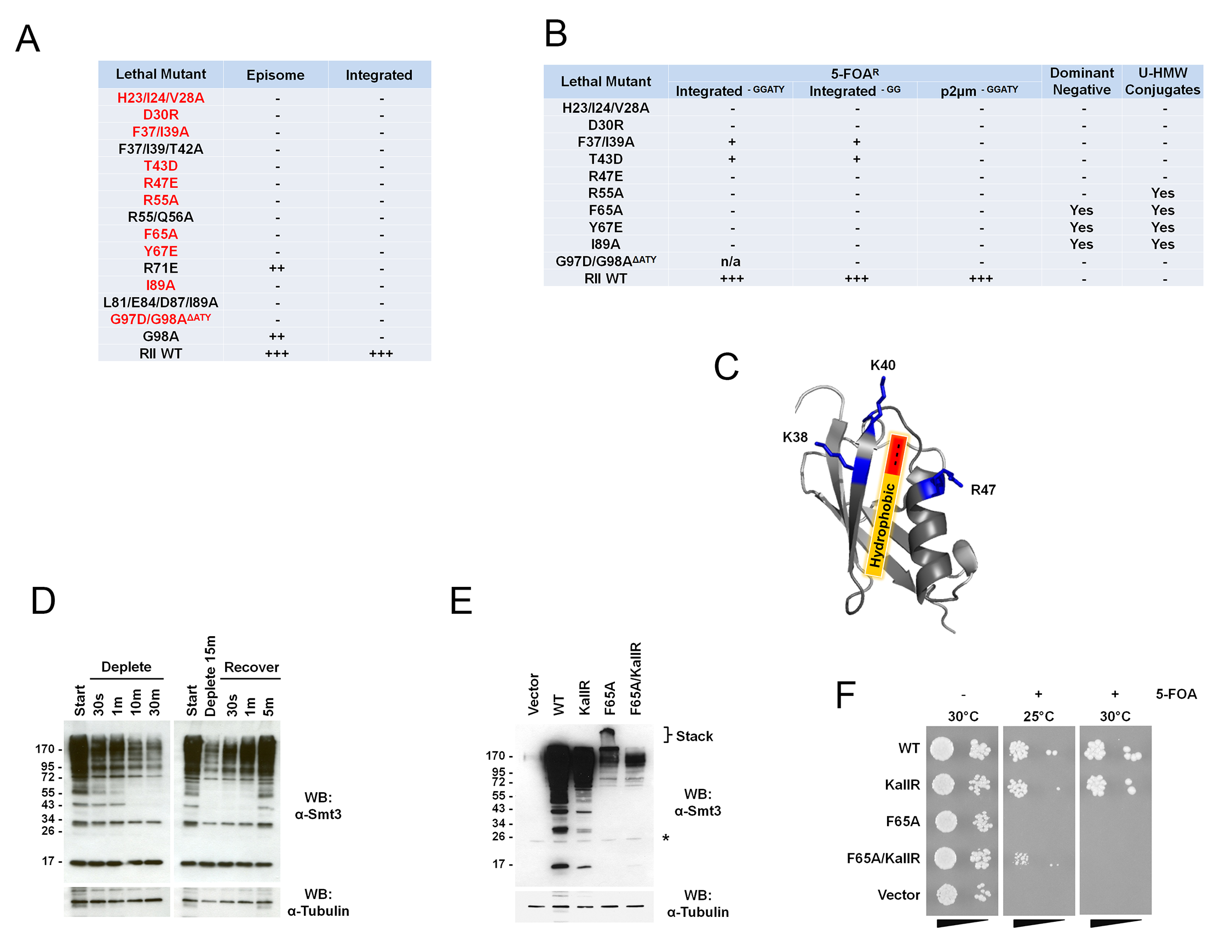

Supplement: S1 Fig — (A) Summary of lethal alleles that failed to complement growth of the smt3Δ strain. Alleles in red failed to suppress both when expressed as episomal and integrated constructs. Multi-site mutations redundant with already represented alleles were not considered for further analysis. Growth after 3 days at 30°C is indicated on a four point scale ranging from no growth (-) to growth similar to wild-type SMT3 (+++). (B) Summary of lethal alleles that were tested for complementation in the smt3Δ shuffle strain to determine if maturation or overexpression of the alleles from a 2 μm plasmid suppressed lethality. Growth after 3 days at 30°C is indicated on a four point scale as in panel A. Each mutant was also expressed in a wild-type SMT3 strain to determine if any produced a dominant negative phenotype. The Smt3 profiles of each mutant expressed in a SUMO1 strain were also analyzed by immunoblotting to detect the presence of ultra-high molecular mass Smt3 conjugates running in the stacking gel. (C) Schematic representing the Smt3 SIM binding surface bound to a canonical SIM. (D) Wild-type SMT3 cells were grown to mid-log phase at 30°C and treated with ATP depletion media for various time points, as indicated. Cells were also allowed to recover for various times after ATP depletion, as indicated. (E) A SUMO1 expressing strain was transformed with the indicated plasmid constructs. Transformants were grown to mid-log phase at 30°C in SC-His and whole cell lysates were analyzed by immunoblotting with a Smt3 antibody. The stacking portion of the gel was left intact so that ultra-high molecular mass conjugates could be visualized. (F) The smt3Δ shuffle strain harboring wild-type SMT3 on a URA3-based plasmid was transformed with the indicated plasmid constructs. The transformants were grown at 25°C on SC–Ura–His overnight and serial dilutions were spotted onto SC–Ura–His and SC–His + 100 μg/ml 5-FOA at 25°C and 30°C to monitor growth in the absence of wild-type SMT3. (TIF) [file pgen.1006612.s001.tif]

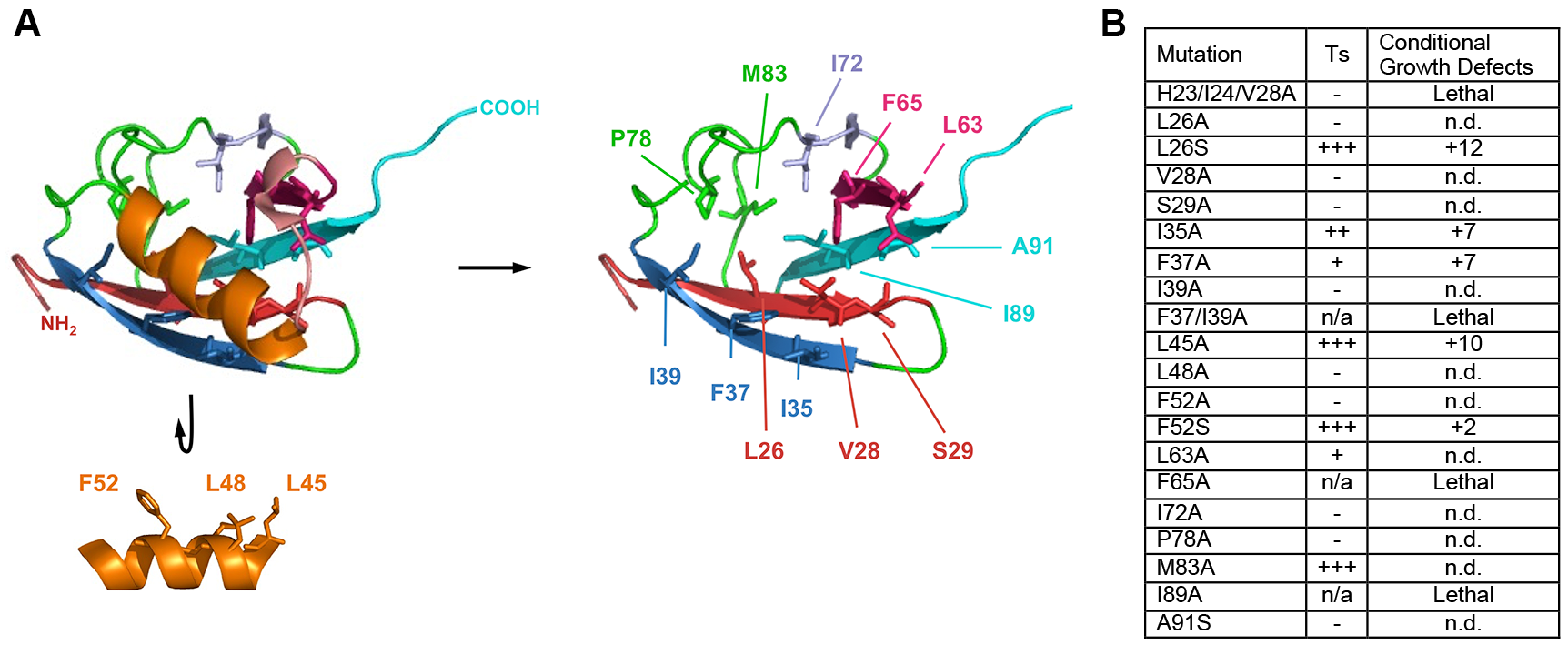

Supplement: S2 Fig — (A) Illustrations of the Smt3 structure highlighting the positions of core residues shielded from solvent. (B) Table summarizing the individual core mutants analyzed, their sensitivity to growth at 39°C (Ts) and the number of additional conditional growth defects detected for each mutant (see Fig 4D for a summary of specific conditional growth defects). n/a = not applicable, n.d. = none detected. (TIF) [file pgen.1006612.s002.tif]

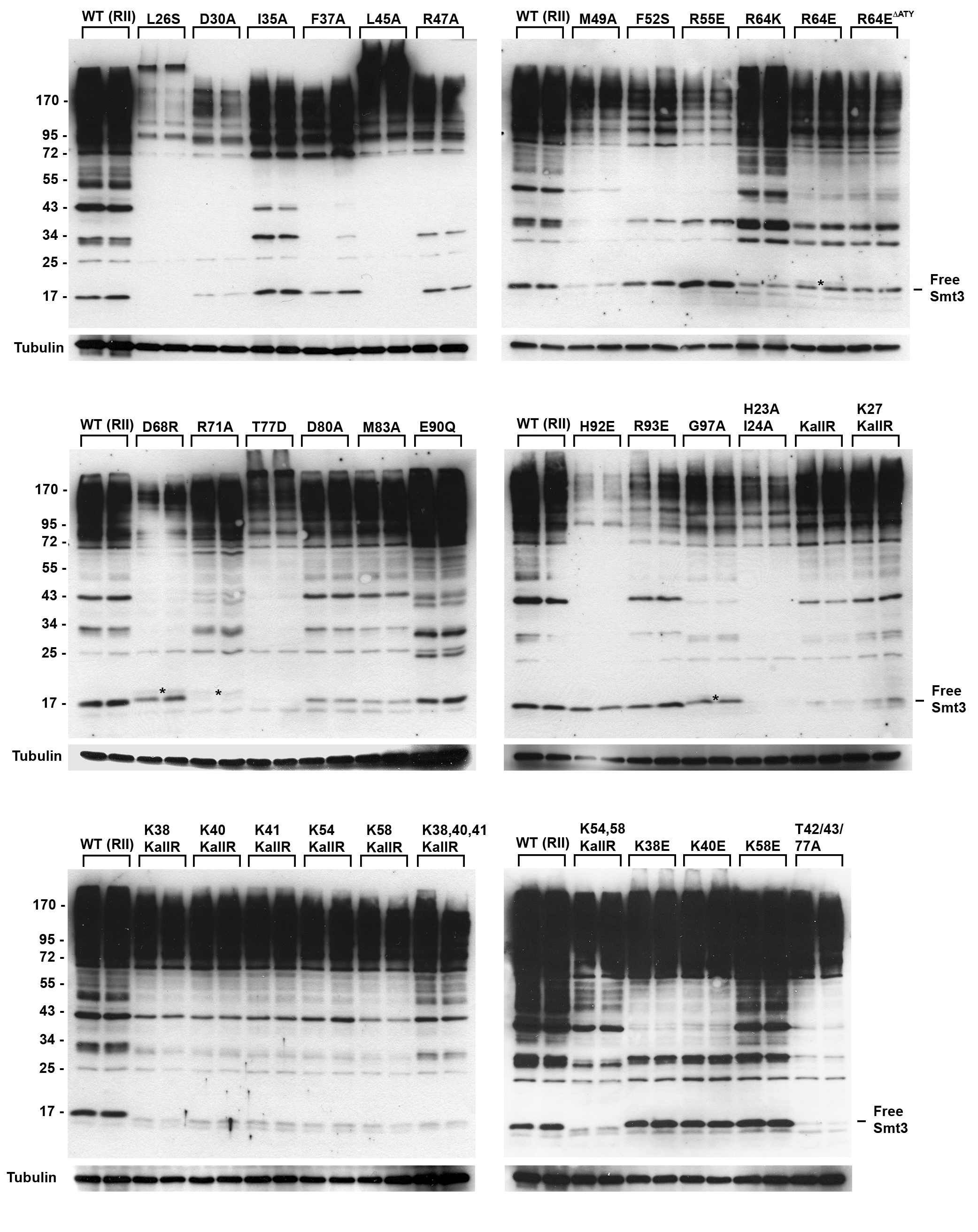

Supplement: S3 Fig — Two independent isolates of the indicated conditional mutant strains were cultured at 30°C. Cells were isolated at mid-log phase and analyzed by immunoblot analysis with an antibody specific for Smt3. Tubulin was also detected as a loading control. Asterisks indicate detection of unprocessed precursor protein in R64E, D68R, R71A and G97A strains. (TIF) [file pgen.1006612.s003.tif]
