# Supplementary material for: A high throughput mutagenic analysis of yeast sumo structure and function
Source: PLoS Genet. 2017 Feb 6;13(2):e1006612. doi: 10.1371/journal.pgen.1006612 (PMC5319795; doi:10.1371/journal.pgen.1006612)
Supplement: S1 Table — Levels of detected Smt3 or Smt3 conjugates are indicated on a scale from undetectable (-) to ultra-high (++++). (PDF) [file pgen.1006612.s004.pdf]

Conditional Allele	Structure-Based Smt3 Interactions	Mature Free Form	Precursor Form	Conjugates	Ultra HMW Forms
**WT**	-	+++	-	+++	-
**L26S**	Smt3 Core	-	-	+	+
**D30A**	E2	+	-	+	-
**I35A**	Smt3 Core/SIM	+++	-	++	-
**F37A**	Smt3 Core/SIM	++	-	++	-
**L45A**	Smt3 Core	-	-	++	+++
**R47A**	SIM	++	-	++	-
**M49A**	-	+	-	+	-
**F52S**	Smt3 Core	+++	-	+	-
**R55E**	SIM	++++	-	+	-
**R64K**	Ulp1	++	-	++++	+
**R64E**	Ulp1	++	+	++	-
**R64E/ATYdel**	Ulp1	++	-	++	-
**D68R**	E2/Ulp1	++	+	+	-
**R71A**	Ulp1	+	+	+++	-
**T77D**	-	-	-	+	++
**D80A**	-	++	-	+++	-
**M83A**	Smt3 Core	++	-	+++	-
**E90Q**	E2/Ulp1	+++	-	++++	-
**H92E**	E2/Ulp1	+++	-	+	-
**R93E**	Ulp1	+++	-	+	-
**G97A**	Ulp1	++	+	+	-
**H23/I24A**	-	-	-	+	-
**KallR**	-	+	-	++	-
**K27/KallR**	E2	+	-	++	-
**K38/KallR**	SIM	+	-	++	-
**K40/KallR**	SIM	+	-	++	-
**K41/KallR**	SIM	+	-	++	-
**K54/KallR**	-	+	-	++	-
**K58/KallR**	-	+	-	++	-
**K38_40_41/KallR**	SIM	+	-	++	-
**K54_58/KallR**	-	+	-	++	-
**K38E**	SIM	+++	-	++	+
**K40E**	SIM	+++	-	++	+
**K58E**	-	++++	-	+++	-
**T42/43/77A**	SIM	+	-	++	-

Table S1. Summary of conditional Smt3 mutant interaction defects and SDS-PAGE/western blot analysis. Levels of detected Smt3 or Smt3 conjugates are indicated on a scale from undetectable (-) to ultra-high (++++).
