# Supplementary material for: A high throughput mutagenic analysis of yeast sumo structure and function
Source: PLoS Genet. 2017 Feb 6;13(2):e1006612. doi: 10.1371/journal.pgen.1006612 (PMC5319795; doi:10.1371/journal.pgen.1006612)
Supplement: S2 Table — The estimated effect of single point mutations on Smt3 thermodynamic stability (ΔΔG) was determined using the Rosetta macromolecular modeling software. Average ΔΔG values, determined as described in the Materials and Methods, are shown for each mutant. (PDF) [file pgen.1006612.s005.pdf]

Mutation	Average ΔΔG
P20A	0.433021257
P20V	0.610269222
E21A	0.557708328
E21Q	1.357576306
E21R	0.525098805
T22A	0.812984152
T22D	0.506448834
H23A	0.894988735
H23E	0.671045304
I24A	2.524607582
N25B	0.191812929
L26A	3.922847235
L26S	3.284598531
K27A	0.948304344
K27E	3.310817909
K27Q	3.345298017
K27R	2.869199636
V28A	2.521066597
S29A	0.946003342
S29D	4.5438594
D30A	3.426857
D30N	0.567945583
D30R	6.946314483
S32A	0.725436024
S32D	2.03291708
S33A	0.422006321
S33D	1.040581929
E34A	-2.526692568
E34Q	1.72455051
E34R	-0.693447808
I35A	1.671658994
F36A	-0.003816363
F37A	4.865410449
K38A	1.384046656
K38E	-0.339235951
I39A	2.669448998
K40A	-0.055093214
K40E	0.562585874
K40Q	0.889270456
K40R	0.874255019
K41A	2.091255214
K41E	2.788827482
K41Q	1.857137376
K41R	-0.455767377
T42A	0.910051836
T42D	0.283202181
T43A	-0.539581385
T43D	1.186834875
P44A	1.376684201
P44V	2.425983354
L45A	2.200446499
R46A	1.502061062
R46E	1.935162323
R46K	1.283583391
R47A	-0.373835265
R47E	-0.396615609
R47K	-0.358936944
L48A	0.722369919
M49A	0.974038479
E50A	2.188423737
E50Q	2.067833066
E50R	3.491220145
A51S	0.732085029
F52A	3.44674734
F52S	4.371642772

**A53S**	2.070332209
**L54A**	0.450367788
**L54E**	0.636691599
**L54Q**	0.581468862
**L54R**	0.796012466
**R55A**	-1.298111888
**R55E**	-0.895411016
**R55K**	-0.556854754
**Q56A**	-0.479384884
**G57A**	2.173250614
**K58A**	1.188066292
**K58E**	1.452481245
**K58Q**	0.271910548
**K58R**	0.997961439
**E59A**	0.025541957
**E59Q**	0.373746695
**E59R**	0.339545487
**M60A**	0.183751003
**D61A**	0.416114701
**D61N**	-0.292328675
**D61R**	0.790033008
**S62A**	0.590499889

**S62D**	1.153496086
**L63A**	2.543905168
**R64A**	-0.33345599
**R64E**	-0.811350798
**R64K**	-0.268132457
**F65A**	5.518837321
**L66A**	0.882541098
**Y67E**	2.773616902
**Y67F**	-0.219549137
**D68A**	0.760262169
**D68N**	-0.446590967
**D68R**	-0.989363444
**G69A**	6.553560756
**I70A**	-0.486045685
**R71A**	0.010803205
**R71E**	-0.77896878
**R71K**	-0.37496777
**I72A**	2.613591277
**Q73A**	0.452049416
**A74S**	1.309925937
**D75A**	19.67454107
**D75N**	22.58764959

**D75R**	7.060811588
**Q76A**	1.330002205
**T77A**	1.780032705
**T77D**	5.947677505
**P78A**	1.885521318
**P78V**	27.74783226
**E79A**	-0.377225821
**E79Q**	0.002642893
**E79R**	0.548958947
**D80A**	0.8316591
**D80N**	2.800465428
**D80R**	2.161902758
**L81A**	1.141273455
**D82A**	0.902796187
**D82N**	-0.693417308
**D82R**	0.546808408
**M83A**	3.133190826
**E84A**	0.482244986
**E84Q**	-0.204085427
**E84R**	-1.640621741
**D85A**	0.762099226
**D85N**	1.105645607

**D85R**	2.444823431
**N86A**	1.927582088
**D87A**	-0.650521171
**D87N**	6.844519298
**D87R**	9.829484155
**I88A**	1.12533826
**I89A**	4.706068412
**E90A**	1.672525852
**E90Q**	0.858412059
**E90R**	2.475634332
**A91S**	0.427371019
**H92A**	1.79793289
**H92E**	1.923991074
**R93A**	-0.426102619
**R93E**	-0.679239524
**R93K**	-0.318237633
**E94A**	-0.533451254
**E94Q**	0.258297048
**E94R**	0.51975834
**Q95A**	0.836229768
**I96A**	0.60778564
**I96R**	0.066444949
**G97A**	0.828035846
**G98A**	0.619673313

Table S2. Thermodynamic stability predictions for Smt3 single point mutations. The estimated effect of single point mutations on Smt3 thermodynamic stability ($\Delta\Delta G$) was determined using the Rosetta macromolecular modeling software. Average $\Delta\Delta G$ values, determined as described in the Materials and Methods, are shown for each mutant.
